# Evolutionary instability of CUG-Leu in the genetic code of budding yeasts

**DOI:** 10.1038/s41467-018-04374-7

**Published:** 2018-05-14

**Authors:** Tadeusz Krassowski, Aisling Y. Coughlan, Xing-Xing Shen, Xiaofan Zhou, Jacek Kominek, Dana A. Opulente, Robert Riley, Igor V. Grigoriev, Nikunj Maheshwari, Denis C. Shields, Cletus P. Kurtzman, Chris Todd Hittinger, Antonis Rokas, Kenneth H. Wolfe

**Affiliations:** 1 Conway Institute and School of Medicine, University College Dublin, Dublin 4, Ireland; 2 Department of Biological Sciences, Vanderbilt University, Nashville, TN 37235 USA; 3 Guangdong Province Key Laboratory of Microbial Signals and Disease Control, Integrative Microbiology Research Centre, South China Agricultural University, Guangzhou, 510642 China; 4 Laboratory of Genetics, Genome Center of Wisconsin, J.F. Crow Institute for the Study of Evolution, Wisconsin Energy Institute, University of Wisconsin-Madison, Madison, WI 53706 USA; 5 DOE Great Lakes Bioenergy Research Center, University of Wisconsin-Madison, Madison, WI 53706 USA; 6 United States Department of Energy Joint Genome Institute, Walnut Creek, CA 94598 USA; 7 Amyris, 5885 Hollis Street, Suite 100, Emeryville, CA 94608 USA; 8 U.S. Department of Agriculture, Mycotoxin Prevention and Applied Microbiology Research Unit, National Center for Agricultural Utilization Research, Agricultural Research Service, Peoria, IL 61604 USA

## Abstract

The genetic code used in nuclear genes is almost universal, but here we report that it changed three times in parallel during the evolution of budding yeasts. All three changes were reassignments of the codon CUG, which is translated as serine (in 2 yeast clades), alanine (1 clade), or the ‘universal’ leucine (2 clades). The newly discovered Ser2 clade is in the final stages of a genetic code transition. Most species in this clade have genes for both a novel tRNA^Ser^(CAG) and an ancestral tRNA^Leu^(CAG) to read CUG, but only tRNA^Ser^(CAG) is used in standard growth conditions. The coexistence of these alloacceptor tRNA genes indicates that the genetic code transition occurred via an ambiguous translation phase. We propose that the three parallel reassignments of CUG were not driven by natural selection in favor of their effects on the proteome, but by selection to eliminate the ancestral tRNA^Leu^(CAG).

## Abstract (web summary)

The genetic code for amino acids is nearly universal, and among eukaryotic nuclear genomes the only known reassignments are of codon CUG in yeasts. Here, the authors identify a third independent CUG transition in budding yeasts that is still ongoing with alternative tRNAs present in the genome.

## Introduction

In the vast majority of organisms, translation of mRNAs into proteins is carried out according to the standard (‘universal’) genetic code, which assigns each of the 64 possible codons to one of the 20 canonical amino acids or as a stop codon. The genetic code is implemented by tRNAs—the adapter molecules that physically connect amino acids to anticodons—and the aminoacyl tRNA synthetases that charge them. The code was initially suggested to be an immutable ‘frozen accident’, because any change to it would alter the sequences of most proteins and would be lethal or highly disadvantageous^1^. Later discoveries showed that the code is not completely frozen because some codon reassignments have occurred, albeit infrequently, during evolution^2–4^. Deviations from the standard code are most commonly seen in mitochondria, which are susceptible to drift because they have their own ribosomes and tRNAs, and only a few genes^5^. Genetic code changes in nuclear genomes are much rarer, and most of the known examples are reassignments where a former stop codon becomes a sense codon^3–6^.

**Fig. 1:**
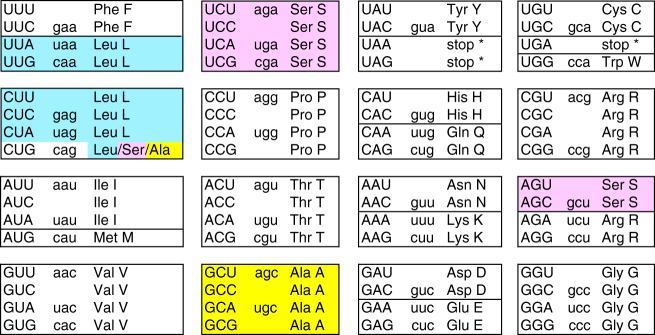
The genetic code. CUG is translated as Leu in the standard code, and as Ser or Ala in the modified codes. Codons are shown in uppercase. The anticodon set of *Saccharomyces cerevisiae* is shown in lowercase, as is tRNA^CAG^, which is not present in *S. cerevisiae*

Genetic code changes in which the meaning of a sense codon is switched from one amino acid to another are particularly rare in nuclear genomes. From 1989 until 2016, the only known example in all eukaryotes was the reassignment of CUG from leucine (its standard meaning) to serine in a clade of budding yeasts that includes *Candida albicans*^7–10^. In 2016, a second reassignment was discovered^11,12^, and surprisingly it also involved reassignment of CUG in a yeast species (*Pachysolen tannophilus*), this time to alanine (Fig. 1).

Theoretical models of how a sense codon could be reassigned from one amino acid to another during evolution can be grouped into two broad categories: ambiguous intermediate models, and unassigned codon models^3^. In the ambiguous intermediate models, a codon becomes reassigned by going through a transition phase during which it can be translated as both the old amino acid and the new one. In their original proposal of this model, Schultz and Yarus^13^ envisaged a cell containing two tRNAs that were charged with different amino acids (alloacceptor tRNAs) but which could both read the same codon, so that the cell produced mixtures of proteins with different translations at each ambiguous site. When it was later discovered that the single tRNA^Ser^ that reads CUG codons in *C. albicans* is mischarged with leucine instead of serine approximately 3% of the time, the ambiguous intermediate model was extended to include ambiguous charging of a single tRNA species^6,14,15^. In the unassigned codon models, one of the 64 codons becomes untranslatable because its tRNA has been lost from the genome, or at least lost its function^3^. The codon can later be captured by another amino acid if a tRNA for that amino acid mutates so that it can read the unassigned codon.

Recently, a ‘tRNA loss-driven’ model of genetic code change was proposed^6,11,16^. In this model, a codon becomes ‘free’ because the tRNA that previously read it has been lost. Translation of the free codon is therefore disturbed or abolished^6^, but the loss of the original tRNA may be partially compensated by wobble decoding of the free codon by other tRNAs, including ones for other amino acids^16^. Depending on the extent to which the free codon is translatable by alloacceptor tRNAs, the tRNA loss-driven model can be regarded as a variant of the unassigned codon model (if there is no translation) or of the ambiguous intermediate model (if alloacceptor tRNAs could read the codon by wobble, even before the original tRNA was lost). Previous studies, both by experimentation and by comparative genomics, have shown that mutations in the anticodon of tRNA genes occur frequently^17,18^. These anticodon shifts can be synonymous, altering the balance between isoacceptor tRNAs for the same amino acid, or nonsynonymous, redeploying the tRNA to a codon for a different amino acid and so causing mistranslation^18^. Many tRNAs are encoded by multigene families, so a mutation in the anticodon of one tRNA gene in a family will often not abolish the organism’s ability to translate the original codon.

The discovery of the CUG-Ala genetic code in *P. tannophilus*^11,12^, and its phylogenetic closeness to the well-known CUG-Ser code in *Candida*, motivated us to investigate the phylogenetic relationship among yeasts with standard and non-standard genetic codes^8,19^. Using whole-genome data to establish phylogeny, and mass spectrometry to determine genetic codes, we show that the CUG codon was reassigned on three separate occasions during the evolution of budding yeasts. We identify a new clade, CUG-Ser2, that transitioned from CUG-Leu to CUG-Ser translation, independently of the similar transition that occurred in the *Candida* clade. We discuss the mechanism of genetic code change, and the cause of the evolutionary instability of CUG-Leu translation in budding yeasts.

## Results

### Phylogeny and genetic code determination

**Fig. 2:**
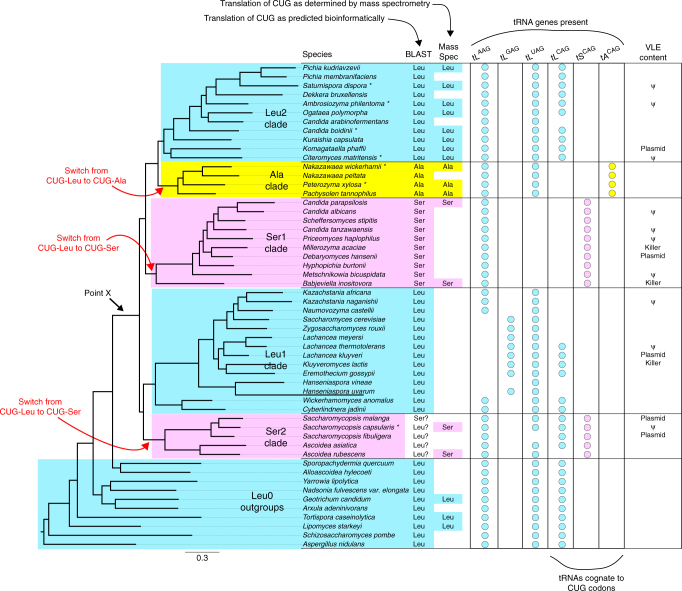
Phylogenomic tree and CUG decoding in 52 yeast species. Blue, pink and yellow indicate CUG translation as Leu, Ser, and Ala, respectively. Point X indicates the last common ancestor of the clades with altered genetic codes. Circles indicate the presence of tRNA genes with the indicated anticodons. The VLE content column shows species where a characterized Virus-Like Element with killer activity is present (Killer), a VLE-like plasmid is present but killer activity has not been demonstrated (Plasmid), or VLE-like pseudogenes are present in the nuclear genome (ψ). Asterisks beside species names indicate genomes sequenced in this study. The tree was constructed from 1237 proteins by maximum likelihood

To identify species with modified genetic codes, we systematically examined the genomes of 52 yeast species (including 7 newly sequenced) and two outgroups. The species phylogeny was inferred by maximum likelihood from whole-genome amino acid data under a site-homogeneous model (Fig. 2). An almost identical tree containing the same set of five clades was obtained using a site-heterogeneous model (Supplementary Fig. 1).

We initially used a BLAST-based method to make a bioinformatic prediction of the genetic code in each species, and then empirically determined the codes of 18 species at key phylogenetic positions by liquid chromatography-tandem mass spectrometry (LC–MS/MS). The data analysis pipeline is summarized in Supplementary Fig. 2. It should be noted that although the LC–MS/MS experiments enable us to identify with confidence the major translation product of CUG codons in each species analyzed (Supplementary Table 1), they do not rule out the possibility of low-level incorporation of other amino acids. Detailed LC–MS/MS and BLAST results for each species are shown in Supplementary Data 1. Examples of LC–MS/MS spectra for species with non-standard codes are shown in Supplementary Fig. 3. The mass error values of CUG-translated residues were similar to those of other residues (Supplementary Fig. 4).

### Extents of the clades with non-standard genetic codes

Examining the BLAST and mass spectrometry results in the context of the inferred species phylogeny, we conclude that there are five monophyletic groups (clades) that differ in their translations of CUG, which we refer to as the Ala, Ser1, Ser2, Leu1, and Leu2 clades (Fig. 2), as well as paraphyletic outgroup taxa (Leu0) with the standard code. The Ala clade contains the genera *Nakazawaea* and *Peterozyma*, as well as *Pachysolen*^11,12^. The split between the Ala and Leu2 clades forms a deep division within the yeast family Pichiaceae^20^. The Leu2 clade includes *Citeromyces* and *Kuraishia*, as well as the industrial yeasts *Komagataella*, *Ogataea*, and *Pichia*. The Ser1 clade contains many pathogenic *Candida* species and extends as far as *Babjeviella*^12^, whose code we confirmed by LC–MS/MS. The newly identified Ser2 clade contains only the genera *Ascoidea* and *Saccharomycopsis*. Ser2 clade species have few CUG codons in conserved genes and gave conflicting results in the BLAST analysis, but the mass spectrometry data showed that CUG is translated as serine in the two species analyzed, *Saccharomycopsis capsularis* and *Ascoidea rubescens* (Fig. 2; Supplementary Table 1). The Ser2 clade is sister to the Leu1 clade, which contains *Saccharomyces cerevisiae* and extends as deep as *Cyberlindnera* and *Wickerhamomyces* (families Saccharomycetaceae, Saccharomycodaceae, and Phaffomycetaceae^20^).

The branches separating the clades with different codes are short, so we evaluated the support for alternative topologies using the Shimodaira-Hasegawa and Approximately Unbiased bootstrapping tests (Supplementary Table 2). These analyses rejected the possibility that the Ser1 and Ser2 clades shared a common ancestor after they diverged from the Ala, Leu1, and Leu2 clades. They also rejected the hypothesis that the Leu1 and Leu2 clades are sisters. Therefore, the most parsimonious explanation of the data is that the CUG codon has been reassigned three times, on three separate branches of the Saccharomycotina tree: once from Leu to Ala, and twice from Leu to Ser (Fig. 2).

### Separate sources of the two *tS*^*CAG*^ genes

**Fig. 3:**
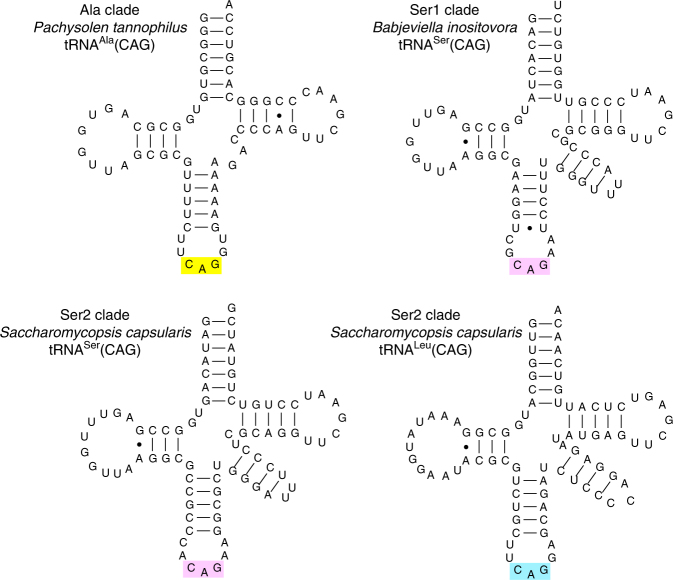
Cloverleaf structures of representative CUG-decoding tRNAs from the Ala, Ser1, and Ser2 clades

Analysis of the tRNA gene sets of each genome shows that the Ser1, Ser2, and Ala clade species each use a different novel tRNA with anticodon CAG to translate CUG codons^11,21^ (Fig. 3; Supplementary Figs. 5–7). The genes for these novel tRNAs were formed by mutating the anticodons of pre-existing tRNA^Ser^ or tRNA^Ala^ genes. Because these pre-existing ‘source’ genes were members of multigene families, the ability of the organism to translate the original codon that they recognized was not lost. The *tS*^*CAG*^ gene (i.e., the gene for tRNA^Ser^ with anticodon CAG) of the Ser2 clade was derived from a different source gene than the *tS*^*CAG*^ gene of the Ser1 clade, which supports the phylogenomic evidence (Fig. 2) that the Leu → Ser reassignments in the Ser1 and Ser2 clades were separate events. Specifically, the novel *tS*^*CAG*^ genes in the two clades are derived from source genes that read the two different serine codon boxes (Fig. 1). In the Ser2 clade, the novel *tS*^*CAG*^ was formed by mutating one of the *tS*^*GCU*^ genes for the tRNA that reads the two AGY serine codons, whereas in the Ser1 clade the novel *tS*^*CAG*^ was formed by mutating one of the *tS*^*AGA*^ or *tS*^*UGA*^ genes for a tRNA that reads some of the four UCN serine codons. The AGY- and UCN-decoding tRNA^Ser^ molecules are distinct and form separate clades in phylogenetic analysis (Supplementary Figs. 6–8; Supplementary Note 1). Notably, the tRNA^Ser^(CAG) molecules of Ser2 clade species contain an A at position 37 which is immediately 3′ of the anticodon (Fig. 3). In *C. albicans* in the Ser1 clade, a G at this position is responsible for the observed 3% mischarging of its tRNA^Ser^(CAG) with leucine^14^, and G_37_ is conserved among all the Ser1 clade species, we analyzed except *B. inositovora* (Fig. 3; Supplementary Fig. 6).

### Retention of *tL*^*CAG*^ as well as *tS*^*CAG*^ in the Ser2 clade

Surprisingly, the genome sequences of four of the five Ser2 clade species indicate that they have a *tL*^*CAG*^ gene as well as a *tS*^*CAG*^ gene (Fig. 3; Supplementary Fig. 9; Supplementary Note 2). In other words, they have two different tRNAs capable of reading CUG: a tRNA^Leu^ and a tRNA^Ser^. To our knowledge, there is no precedent for a genome that naturally produces competing tRNAs that read the same codon but insert different amino acids. This suggests that Ser2 clade species might produce ‘statistical proteins’ with a mix of serine and leucine incorporation at CUG sites^22,23^, but in our mass spectrometry analysis (of *Saccharomycopsis capsularis*) we found only robust evidence for translation of CUG as serine (Supplementary Table 1). We also found that *tS*^*CAG*^ but not *tL*^*CAG*^ is transcribed in two *Saccharomycopsis* species when grown in the same conditions (YPD media) as were used for mass spectrometry (Supplementary Fig. 10). However, the *tL*^*CAG*^ gene is conserved among the three *Saccharomycopsis* species sequenced, whereas its flanking DNA has diverged, so it is unlikely to be a pseudogene (Supplementary Fig. 11). It is also syntenic with the functional *tL*^*CAG*^ gene in the Leu1 clade (Supplementary Fig. 9).

### Selection against tRNA^Leu^(CAG) in the Leu1 and Leu2 clades

What made the CUG codon so unstable in yeasts? The meaning of sense codons in nuclear genes has been stable throughout all of eukaryotic evolution^2–6^, except in the three yeast clades where CUG became reassigned. The observations that three reassignments occurred independently in three closely related eukaryotic lineages, and that they all involved the same codon, strongly suggest that the reassignments shared a common evolutionary cause. As described below, we hypothesize that the shared cause was natural selection acting against the ancestral tRNA^Leu^(CAG), and that the selective pressure was caused by a killer toxin that attacked this specific tRNA molecule (see Discussion).

**Fig. 4:**
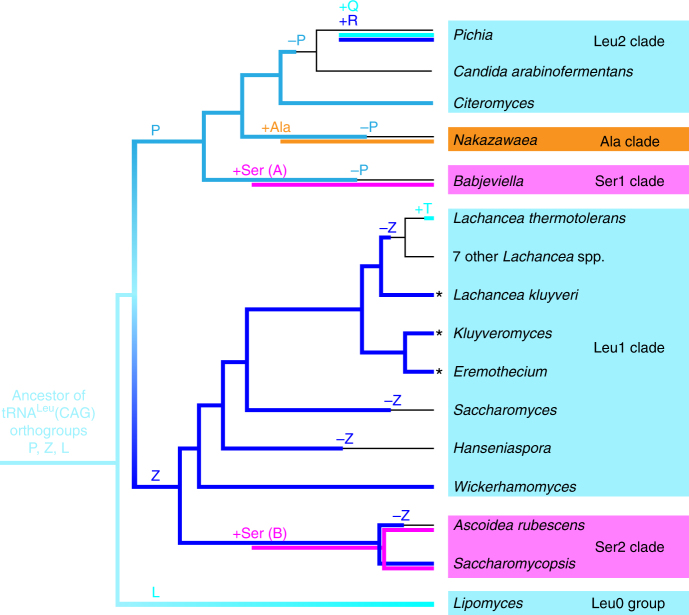
Summary of evolutionary losses and gains of tRNAs with CAG anticodons. Colored branches on the tree indicate presence of tRNA^Leu^ (blue), tRNA^Ala^ (orange), and tRNA^Ser^ (pink) molecules, with shades of blue indicating different tRNA^Leu^ orthogroups (designated *P*, *Z*, *L*, *Q*, *R*, and *T*; Supplementary Fig. 9; Supplementary Note 3). Plus and minus symbols indicate inferred gains and losses of tRNA types. For each branch, only some representative genera or species are named. Asterisks indicate taxa with large introns in *tL*^*CAG*^*-Z*. Species with no tRNA^CAG^ gene (thin lines) translate CUG as Leu by wobble using tRNA^Leu^(UAG)

The primary evidence for selection against tRNA^Leu^(CAG) is that it has been lost at least seven times during budding yeast evolution (Fig. 4), in stark contrast to the general stability of tRNA anticodon repertoires in yeasts^16^. Three losses of tRNA^Leu^(CAG) occurred in the clades that changed their genetic codes, and were facilitated by the prior emergence of tRNA^Ser^(CAG) or tRNA^Ala^(CAG) tRNAs to decode CUG. Four additional losses of ancestral *tL*^*CAG*^ genes occurred in the Leu1 and Leu2 clades, without changing their genetic codes (Fig. 4, ‘–Z’ and ‘–P’ symbols). The sets of tRNAs that species in these clades now use to translate CUN as leucine show three unusual features that are consistent with an hypothesis of selective pressure against the ancestral tRNA^Leu^(CAG). These features are summarized here and described in more detail in Supplementary Note 3. First, the *tL*^*CAG*^ gene was modified in some Leu1 clade species by unprecedented expansion of its intron, making it the largest canonical tRNA intron known (134–318 nt; Supplementary Fig. 12). The intron contains extensive secondary structure, which is likely to slow the rate of formation of the mature spliced and base-modified tRNA^Leu^(CAG). Second, the gene was replaced in other Leu1 and Leu2 species (represented by *Pichia* and *Lachancea thermotolerans* in Fig. 4) that eliminated the ancestral gene and later regained paralogous *tL*^*CAG*^ genes, probably by horizontal gene transfer. Third, the gene was eliminated, without replacement, in *Saccharomyces* by losing the standard eukaryotic modification of the wobble base U_34_ in tRNA^Leu^(UAG), allowing this tRNA to read CUG as well as CUA codons. Leu1 clade species also show other unusual deviations from the normal wobble rules used by eukaryotes, in the way they read the CUN codon box (Supplementary Note 3).

## Discussion

The coexistence of ancestral *tL*^*CAG*^ and novel *tS*^*CAG*^ genes in Ser2 clade species provides direct support for the ambiguous intermediate model of genetic code evolution^3,13^. Because the *tL*^*CAG*^ gene in the Ser2 clade is orthologous to the functional *tL*^*CAG*^ gene in the Leu1 clade (Supplementary Fig. 9), the genomic data indicate that the Ser2 clade underwent a transition from CUG-Leu to CUG-Ser translation via an intermediate phase in which both types of tRNA gene were present and functional in the same species. It is evident that *tL*^*CAG*^ was not lost before *tS*^*CAG*^ was gained, because *tL*^*CAG*^ is still present. The Ser2 clade species appear to be in the final stages of the evolutionary transition between genetic codes. Only *Ascoidea rubescens* has completed the transition and lost the *tL*^*CAG*^ gene. In the other four species, the novel tRNA^Ser^(CAG) is now the major tRNA species decoding CUG, but the gene for tRNA^Leu^(CAG) has been conserved even though it was not transcribed or used for translation in our experiments. The evolutionary conservation of *tL*^*CAG*^ in four Ser2 clade species is puzzling, but a possible explanation is that tRNA^Leu^(CAG) may be required for translation of some genes specifically expressed in conditions that we did not examine, for example meiosis. It suggests that some CUG sites in these four species still code for essential leucine residues.

We propose that the genetic code changes were driven by natural selection against the existence of a particular tRNA species (the ancestral form of tRNA^Leu^(CAG)), and not by selection in favor of the proteomic consequences of the code changes. Selection in favor of the proteomic changes seems implausible in view of the phylogenomic evidence that three independent reassignments occurred (Fig. 2), because it is difficult to envisage how the replacement of thousands of leucine residues with both alanine (in one lineage) and serine (in two other lineages) could have had beneficial effects on protein sequences and been advantageous in both situations. In contrast, AlaRS and SerRS are the only two aminoacyl tRNA synthetases that do not require particular bases to be present in the anticodon of the tRNAs they charge^24^, so these are the only two amino acids to which CUG could have readily been reassigned from leucine simply by mutating the anticodon of an existing tRNA^Ala^ or tRNA^Ser^^16^, although multiple mutations are required in the anticodon. Our hypothesis of selection against tRNA^Leu^(CAG) is also consistent with the tendency for CUG codons to be located at non-essential sites in species that have changed their genetic code^9^. CUG codons are relatively rare in the clades that changed codes. They tend to occur in orphan genes, or in regions of genes that do not align well with other species, as opposed to sites that are well conserved (Supplementary Figs. 13, 14). This pattern would not be expected if the genetic code changes were favored because of their effect on protein sequences.

We hypothesize that the agent of selection against tRNA^Leu^(CAG) may have been a Virus-Like Element (VLE). VLEs are cytoplasmic linear DNA plasmids (also called killer plasmids) that code for a toxin and an antitoxin^25,26^. VLEs or VLE-like plasmids are present in 1–2% of budding yeast strains^27^. Cells carrying a VLE secrete a toxin that kills cells, from the same or other species, that lack the VLE. The toxins are ribonucleases that cleave the anticodon loops of specific tRNAs. The two known targets of this class of killer element are tRNA^Glu^(UUC), which is cleaved by a toxin found in some strains of *Kluyveromyces lactis* (Leu1 clade), and tRNA^Gln^(UUG), which is cleaved by toxins from strains of *Millerozyma acaciae* and *Debaryomyces robertsiae* (both Ser1 clade)^25^. Similar, but uncharacterized, VLE-like plasmids have been described in Leu2 and Ser2 clade species^28,29^. BLAST searches show that the nuclear genomes of many species in the Ser1, Ser2, Leu1, and Leu2 clades contain pseudogenes of VLE-like plasmids^30,31^ (Fig. 2; Supplementary Table 3), which indicates that these species have been infected by VLEs in the past. In contrast, VLE-like sequences are absent from the genomes of the Leu0 species, an outcome whose probability is 0.01 under the assumption of uniform distribution of the 18 found VLE-like sequences across the 54 analyzed species.

In our hypothesis, a VLE with a toxin specific for tRNA^Leu^(CAG) infected the common ancestor of five clades (point X in Fig. 2). The infection reduced the availability of tRNA^Leu^(CAG) in the pool of leucine tRNAs, causing selection in favor of alternative ways to read CUG codons without using the susceptible tRNA. Some yeast lineages responded by changing their genetic codes, whereas others altered the sets of tRNA^Leu^ genes they contain and managed to retain the standard code, either by changing their wobble rules or by acquiring versions of tRNA^Leu^(CAG) that were resistant to the toxin. Incompatibilities between different genetic codes may have contributed to reproductive isolation among the clades that emerged shortly after point X. The outcome of the infection resembles the predictions of the ‘tRNA loss-driven’ model^6,11,16^, but the initial event was destruction of tRNA^Leu^(CAG) molecules by the postulated toxin rather than loss of the *tL*^*CAG*^ gene. We cannot tell if the hypothesized VLE still exists or was transient. Stochastic losses of infection could explain how ancestral *tL*^*CAG*^ genes survive in a few species. If our hypothesis is correct, reorganization of the genetic code can be regarded as a radical mechanism of host defense against an infectious agent^32,33^.

## Methods

### Genome sequences

We sequenced and assembled the genomes of seven species using the iWGS pipeline^34^, selecting the assembly with the highest N50: the Leu2 clade species *Saturnispora dispora* (strain NRRL Y-1447, SPAdes assembly), *Ambrosiozyma philentoma* (NRRL Y-7523, SPAdes), *Candida boidinii* (NRRL Y-2332, DISCOVAR), and *Citeromyces matritensis* (NRRL Y-2407, MASURCA); the Ala clade species *Nakazawaea wickerhamii* (NRRL Y-2563, DISCOVAR) and *Peterozyma xylosa* (NRRL Y-12939, DISCOVAR); and the Ser2 clade species *Saccharomycopsis capsularis* (NRRL Y-17639, DISCOVAR).

### Phylogenetic tree construction

The phylogenetic tree in Fig. 2 was constructed^20^ from genomic data for 52 yeast taxa and 2 fungal outgroups, using a set of 1237 genes from BUSCO^35^. Each locus sampled had minimum sequence occupancy ≥27 taxa and sequence length ≥167 amino acid residues. To avoid any influence of mistranslated CUG codons on the phylogenetic tree, amino acids encoded by CUG codons were substituted by “X”. We used RAxML^36^ version 8.2.0 to perform maximum likelihood analyses of the concatenation data matrix (607,754 sites) under an unpartitioned scheme (a LG+GAMMA model) and a gene-based partition scheme (1237 partitions; each has its own model). The two ML trees produced by RAxML were topologically identical and were also found by the program IQ-TREE^37^ version 1.5.1. Branch support for each internode was evaluated with 100 rapid bootstrapping replicates using RAxML^38^. Because running an analysis using the site-heterogeneous CAT model in PhyloBayes is computationally intractable for our concatenated dataset, the C60 model (a maximum likelihood variant of Bayesian CAT model) implemented in IQ-TREE was used to infer ML phylogeny with passing ‘-m C60+LG+G4 -bb’ to specify the site-heterogeneous model and to conduct 1000 ultrafast bootstrap replicates (Supplementary Fig. 1). Running time was ~25 days with 32 CPUs. Phylogenetic trees for tRNA genes were constructed using PhyML after intron removal and MUSCLE alignment^39^. To calculate the probability of all VLEs appearing outside the Leu0 clade under the hypothesis that they are uniformly distributed across the tree, we calculated the number (44 choose 18)/(54 choose 18) which is approximately equal to 1.01%.

### Bioinformatics methods

For bioinformatic inference of genetic codes, to maintain consistency of annotation all genomes were annotated using a simple method that identified all open reading frames (ORFs, the region between one stop codon and the next) as potential genes, provided that they were ≥180 bp long and did not overlap by >50 bp with a longer ORF. ORFs were translated using the standard genetic code and searched by BLASTP against the Yeast Gene Order Browser database of proteins^40^ with a cutoff of *E* ≤ 1e−10. The set of BLAST high-scoring pairs (HSPs) from a genome was then processed to populate a matrix of 64 codons × 20 amino acids (Supplementary Data 2) for the query species, as follows. For every codon site inside a predicted ORF, if there were ≥5 YGOB database proteins aligned against the site, and > 80% of these had the same amino acid at the site, then for every HSP at this site we assigned to the amino acid aligned against that site a score of 1/*n*, where *n* is the total number of proteins aligned against the site (Supplementary Fig. 2). Counts of CUG codon occurrence in ORFs, in genes with HSPs, and in HSP regions (Supplementary Figs. 13, 14) were calculated from the results of BLAST searches against the BUSCO Ascomycota ‘ancestral’ database^35^.

tRNA genes were predicted using tRNAscan-SE^41^, with introns removed by our own Python code. In some *Kluyveromyces* species, the *tL*^*CAG*^ gene was not predicted by tRNAscan-SE due to its unusually long intron but was found by BLASTN. In our phylogenetic trees, tRNAs are identified with names such as S_CAG1_Ser1_Babino_r2_i_25. The fields (separated by underscores) in these names are: inferred amino acid; anticodon; clade; 3-letter genus and species abbreviations; “r” indicates the repeat count of genes coding for identical tRNAs (ignoring introns) in this species; “i” or “n” indicates presence or absence of an intron in the gene; intron length.

To identify pseudogenes of genes from VLEs or VLE-like plasmids (cytosolic linear DNA plasmids without demonstrated killer activity) located in yeast nuclear genomes, we first constructed a database of known yeast VLEs^25,27^ (Supplementary Table 3). Proteins encoded by these elements were used as queries in TBLASTN searches against a database of all 54 fungal genomes, and potential pseudogenes were then tested for reciprocal BLASTX hits to VLE proteins.

### LC–MS/MS

Total protein was extracted from cultures grown in YPD, and analyzed by LC–MS/MS. Triplicate samples were run on a Thermo Scientific Q Exactive mass spectrometer connected to a Dionex Ultimate 3000 (RSLCnano) chromatography system. Tryptic peptides were resuspended in 0.1% formic acid. Each sample was loaded onto a fused silica emitter (75 μm i.d., pulled using a Sutter Instruments P2000 laser puller), packed with 1.8 μm 120 Å UChrom C18 packing material (NanoLCMS Solutions) and was separated by an increasing acetonitrile gradient over 60 min at a flow rate of 250 nL/min. The mass spectrometer was operated in positive ion mode with a capillary temperature of 320 °C, and with a potential of 2300 V applied to the frit. All data were acquired with the mass spectrometer operating in automatic data-dependent switching mode. A high resolution MS scan (300–1600 *m*/*z*; Supplementary Table 4) was performed using the Q Exactive to select the eight most intense ions prior to MS/MS analysis using HCD.

In a first approach to empirical genetic code determination (Supplementary Fig. 2), de novo peptide sequences were extracted from the LC–MS/MS data using PEAKS^42^ Studio 7 software. Settings were Parent Mass Error Tolerance 10.0 ppm, Fragment Mass Error Tolerance 0.03 Da, fixed modifications: carbamidomethylation, variable modifications: oxidation. Peptides that mapped to a unique site in the genome with ≤1 mismatch to the standard-code translation were identified^12^. If a genomic site mapped to multiple peptides, all peptides were required to agree. This method deduced the complete genetic code table of each species (Supplementary Data 3), except for ambiguity of Leu and Ile, which cannot be differentiated by mass, and showed that no species had reassigned any codon other than CUG.

In a second approach (Supplementary Fig. 2), which used peptide mass fingerprinting rather than complete de novo peptide sequences, we generated 19 hypothetical proteome databases from each genome, corresponding to every possible sense translation of CUG^11^. We then used MaxQuant^43,44^ version 1.5.5.1 to identify peptides that had a unique match to only one of these databases, filtered the matches to include only CUG-encoded residues that were individually supported by b- and/or y-ion data, and tabulated the translations of CUG seen at each genomic site (Supplementary Table 1). The accepted mass ranges for individual amino acids in b/y-ion fragment determination are listed in Supplementary Table 5. MaxQuant parameters were set to a false discovery rate of 1% (other parameters are given in Supplementary Data 4).

The nature of the LC–MS/MS experiment does not allow us to directly quantify the levels of (mis)incorporation of different amino acids at any particular CUG site of interest. Because LC–MS/MS involves sampling a limited number of tryptic peptides for fragmentation (an average of 32,051 unique peptides per species in our data; Supplementary Table 4), and these peptides were chosen randomly by the mass spectrometer (data-dependent acquisition mode), in general each genomic CUG site whose translation was detected was spanned by only one peptide. Therefore, we tabulated for each species, for all the CUG sites in its genome that were spanned by a peptide, the proportion of those sites that matched Ser, Ala, Leu, etc., in the peptide. This proportion exceeds 90% for the major translation product of CUG in all species except *C. boidinii* (86%) and *A. rubescens* (80%) (Supplementary Table 1).

### RT-PCR of Ser2 clade *tL*^*CAG*^ and *tS*^*CAG*^ expression

Genomic DNA of *S. capsularis* and *S. malanga* was extracted by homogenization of stationary phase cultures with glass beads followed by phenol-chloroform extraction and ethanol precipitation. RNA was extracted from log-phase cultures by hot acid phenol-chloroform extraction. Following DNase I (Invitrogen) treatment, cDNA was synthesized using random primers (High-Capacity cDNA Reverse Transcription Kit with RNase Inhibitor; Applied Biosystems). Primer sequences are listed in Supplementary Table 6. RT-PCR amplification was performed using GoTaq (Promega) polymerase or Q5 high fidelity polymerase (NEB) for 30 cycles with an annealing temperature of 55 °C.

### Data availability

Genome sequences and raw reads have been deposited in GenBank as BioProject PRJNA386659, under DDBJ/ENA/GenBank accessions NHAL00000000–NHAR00000000. The versions described in this paper are versions NHAL01000000–NHAR01000000. The genome sequences of the other 47 species analyzed are from public sources (Supplementary Table 7). The mass spectrometry proteomics data have been deposited to the ProteomeXchange Consortium via the PRIDE partner repository^45^ with the dataset identifier PXD008827.

## Electronic supplementary material

Supplementary Information

Description of Additional Supplementary Files

Supplementary Data 1

Supplementary Data 2

Supplementary Data 3

Supplementary Data 4
