# Supplementary material for: Evolutionary instability of CUG-Leu in the genetic code of budding yeasts
Source: Nat Commun. 2018 May 14;9:1887. doi: 10.1038/s41467-018-04374-7 (PMC5951914; doi:10.1038/s41467-018-04374-7)
Supplement: Supplementary file 1 — Supplementary Information [file 41467_2018_4374_MOESM1_ESM.pdf]

Supplementary Information

Evolutionary Instability of CUG-Leu in the Genetic Code of Budding Yeasts

Krassowski *et al.*

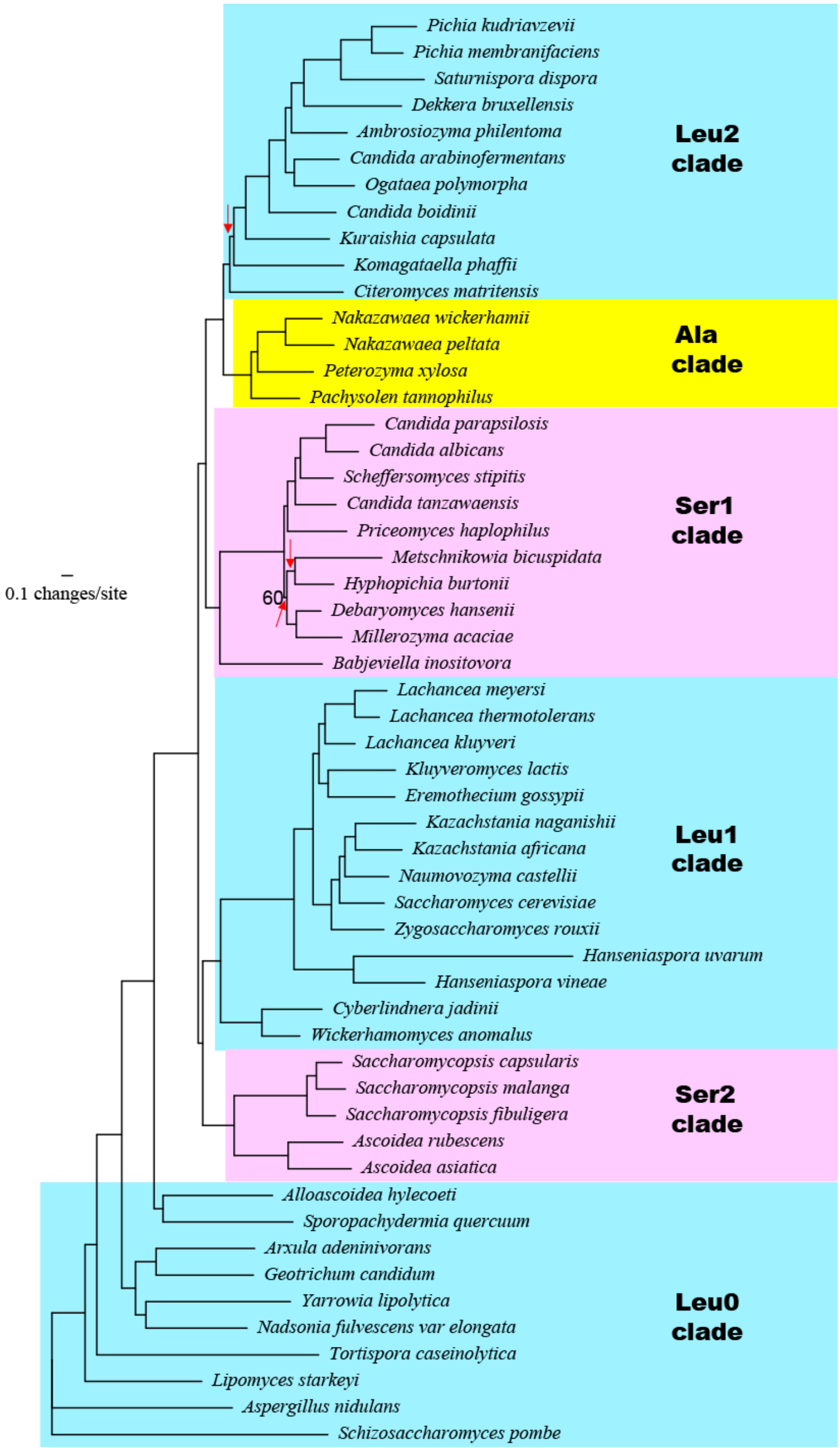

Supplementary Fig. 1. Phylogeny inferred from concatenated amino acid data matrix of 54 taxa and 1,237 genes under the site-heterogeneous ML model C60+LG+G4 implemented in IQ-TREE software. Nodes have 100% bootstrap support unless otherwise noted. Red colored arrows denote branches that conflict with the concatenation-based ML tree under the site-homogeneous model LG+G4 (Fig. 2). None of them occur between different clades. Specifically, two incongruent internodes occur within the Ser1 clade and one within the Leu2 clade.

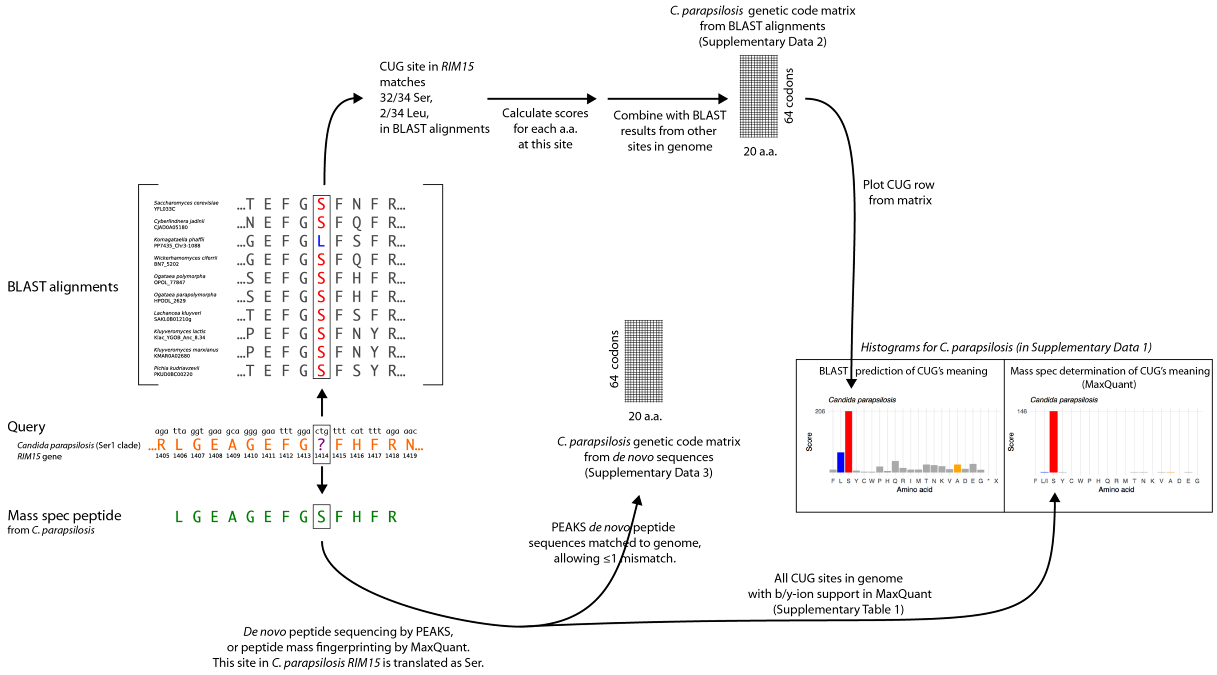

Supplementary Fig. 2. Summary of the methods used for bioinformatic prediction and LC-MS/MS confirmation of genetic codes. The Query line (orange) shows an example of a short section of the *RIM15* gene of *Candida parapsilosis*, which contains a CTG codon at position 1414 (boxed). Above this, BLAST alignments are shown for 10 of the 34 proteins in other species that aligned to it in the BLAST analysis. For the CTG at position 1414, 32 of the aligned proteins contained Ser and 2 contained Leu at this site, resulting in scores of 0.94 (32/34) for Ser and 0.06 (2/34) for Leu for this site in *C. parapsilosis RIM15*. Scores for every codon site in every gene in *C. parapsilosis*, excluding unreliable alignments, were totaled to generate a matrix of 64 codons x 20 amino acids (Supplementary Data 2), which is the bioinformatic prediction of the complete genetic code in *C. parapsilosis*. The row of the matrix corresponding to predicted translations of CUG is plotted as a histogram and shows that Ser (red bar) is the prediction with the highest score. In the lower section, a match between this region of *C. parapsilosis RIM15* and a *C. parapsilosis* peptide identified by mass spectrometry is shown, demonstrating that this CTG site in *RIM15* is translated as Ser. Peptides sequenced *de novo* using PEAKS were processed to compile an empirical 64 x 20 genetic code matrix from all matches between the peptide sequence and the genome (Supplementary Data 3). Peptides identified by mass fingerprinting using MaxQuant, that spanned a CUG site and had b/y-ion support for the amino acid at that site, were compiled (Supplementary Table 1) and CUG translation frequencies plotted as a histogram, showing that Ser (red bar) is the most common translation of CUG sites in detected *C. parapsilosis* peptides. Histograms for all species are in Supplementary Data 1.

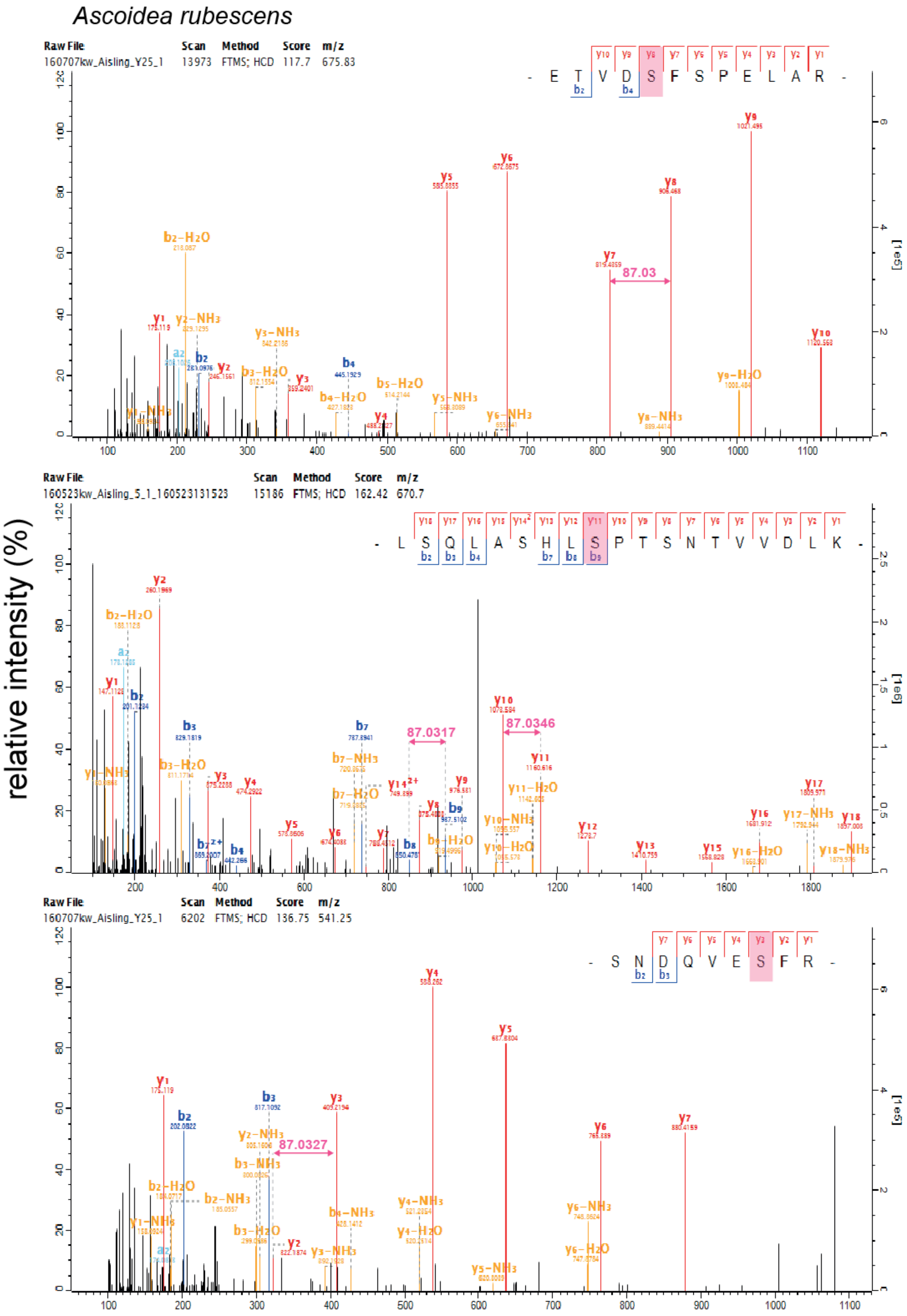

Supplementary Fig. 3a. Representative MS/MS spectra for non-standard genetic codes in *Ascoidea rubescens*. The identified peptide sequences are shown for three spectra, with matched y-ions in red and b-ions in blue. Amino acids translated from CUG are colored pink (serine) or yellow (alanine).

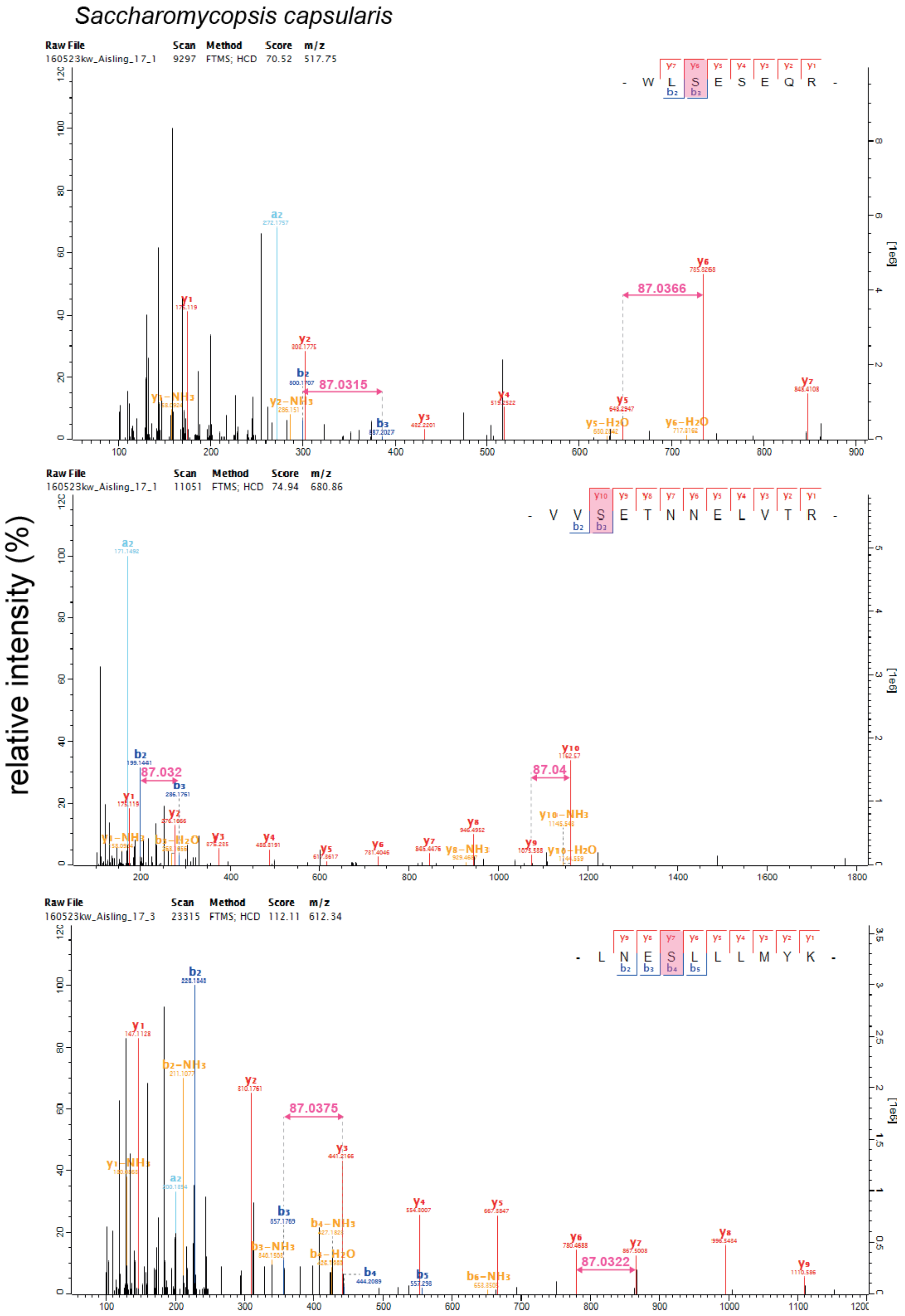

Supplementary Fig. 3b. Representative MS/MS spectra for non-standard genetic codes in *Saccharomycopsis capsularis*. The identified peptide sequences are shown for three spectra, with matched y-ions in red and b-ions in blue. Amino acids translated from CUG are colored pink (serine) or yellow (alanine).

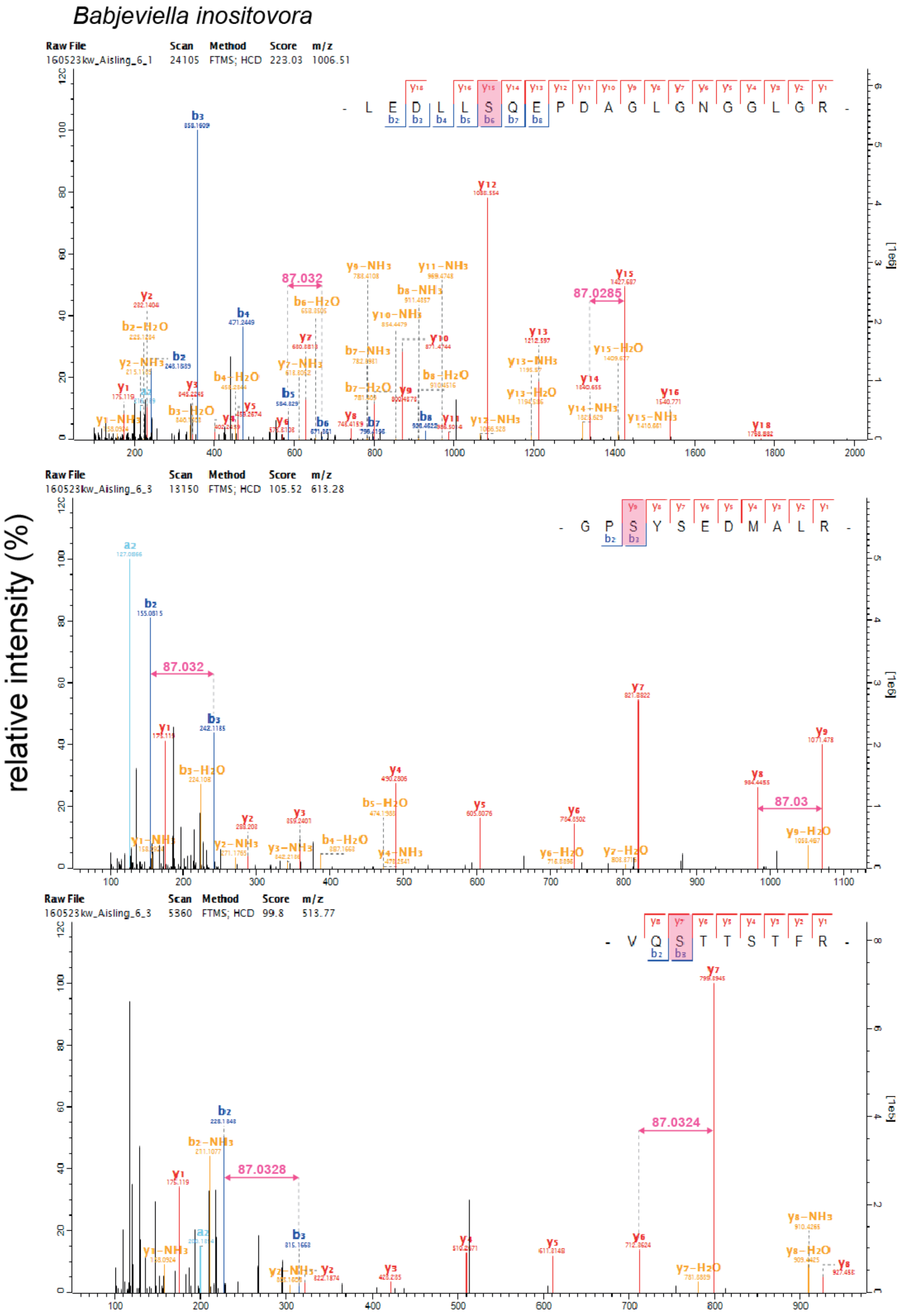

Supplementary Fig. 3c. Representative MS/MS spectra for non-standard genetic codes in *Babjeviella inositovora*. The identified peptide sequences are shown for three spectra, with matched y-ions in red and b-ions in blue. Amino acids translated from CUG are colored pink (serine) or yellow (alanine).

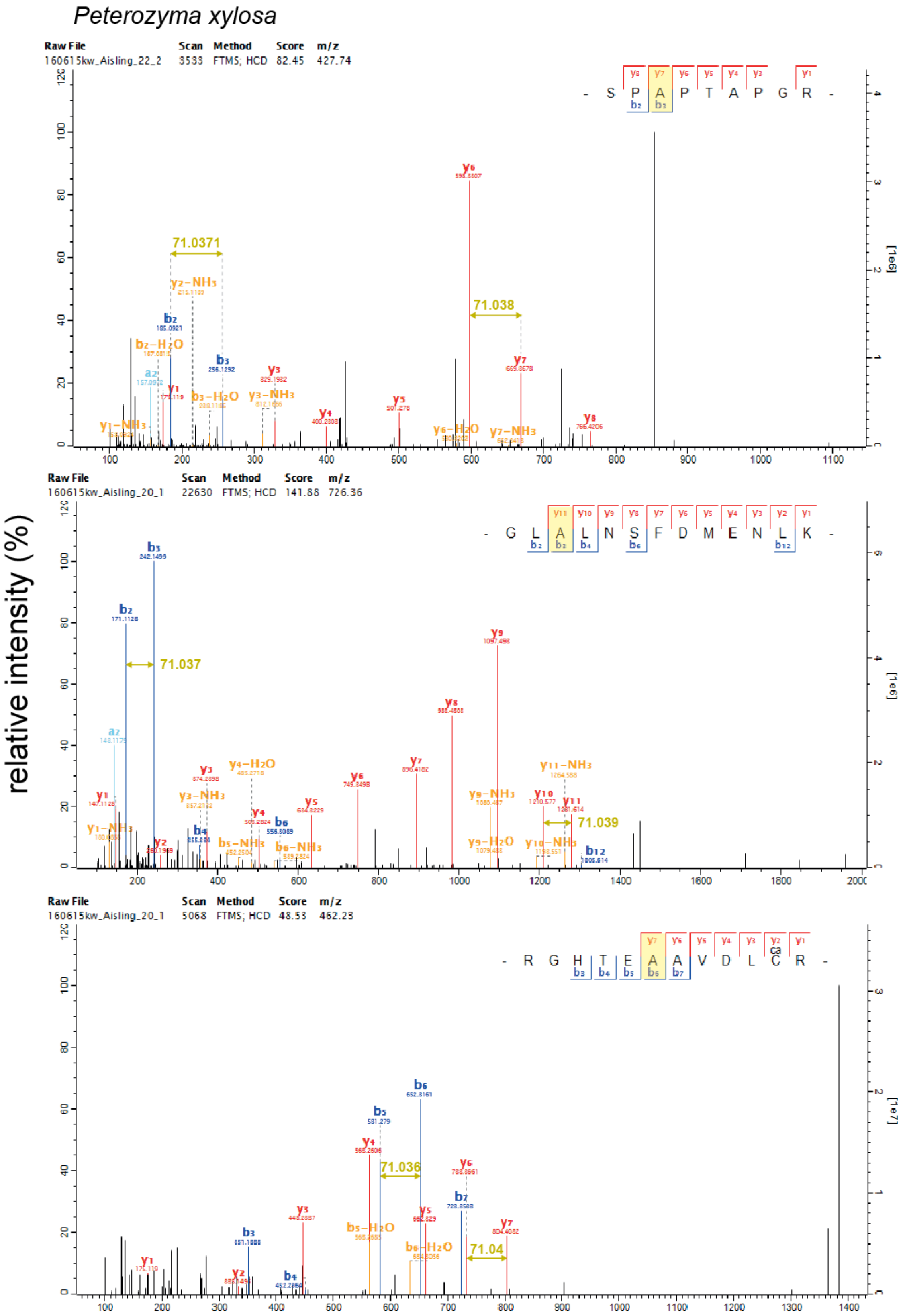

Supplementary Fig. 3d. Representative MS/MS spectra for non-standard genetic codes in *Peterozyma xylosa*. The identified peptide sequences are shown for three spectra, with matched y-ions in red and b-ions in blue. Amino acids translated from CUG are colored pink (serine) or yellow (alanine).

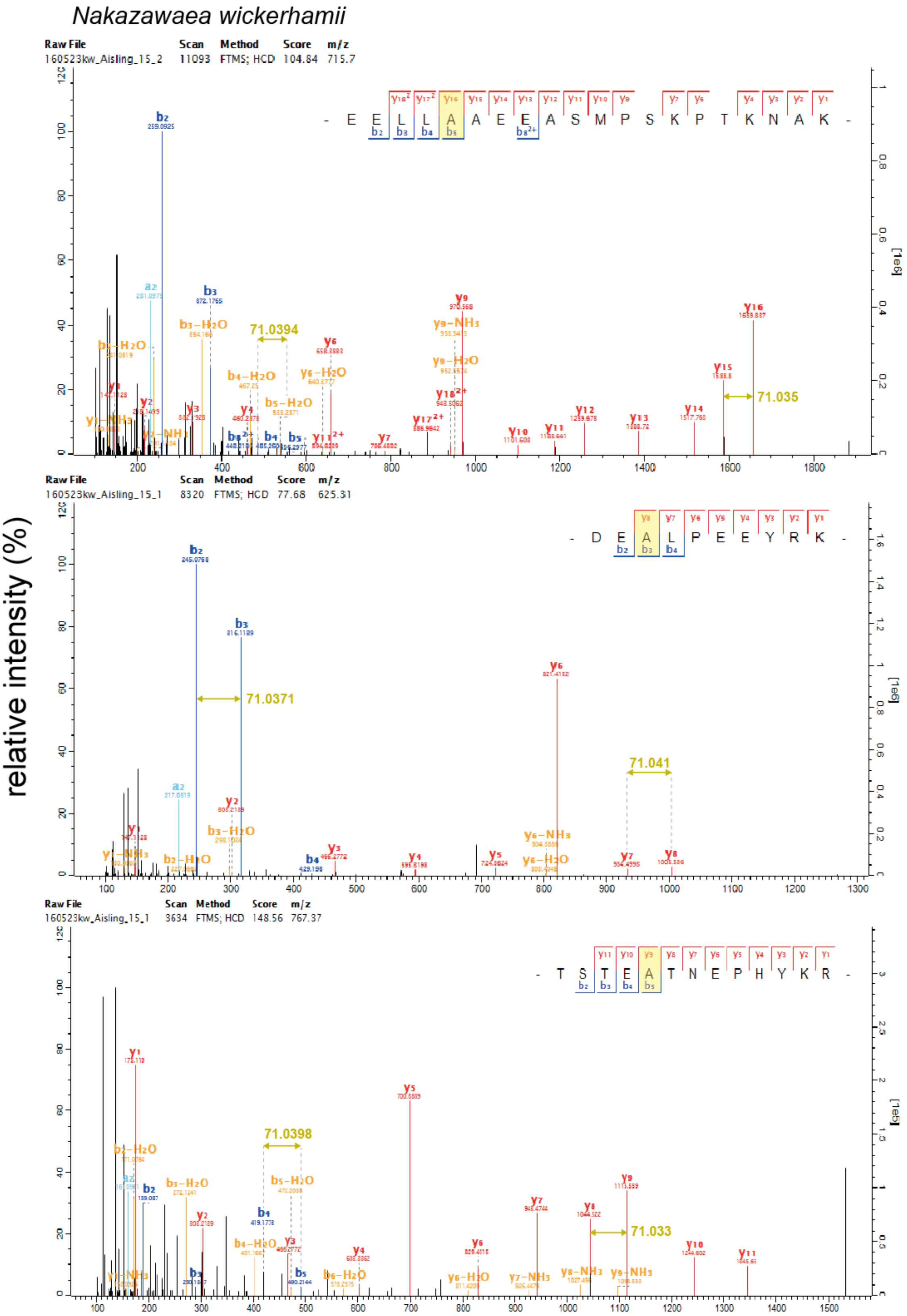

Supplementary Fig. 3e. Representative MS/MS spectra for non-standard genetic codes in *Nakazawaea wickerhamii*. The identified peptide sequences are shown for three spectra, with matched y-ions in red and b-ions in blue. Amino acids translated from CUG are colored pink (serine) or yellow (alanine).

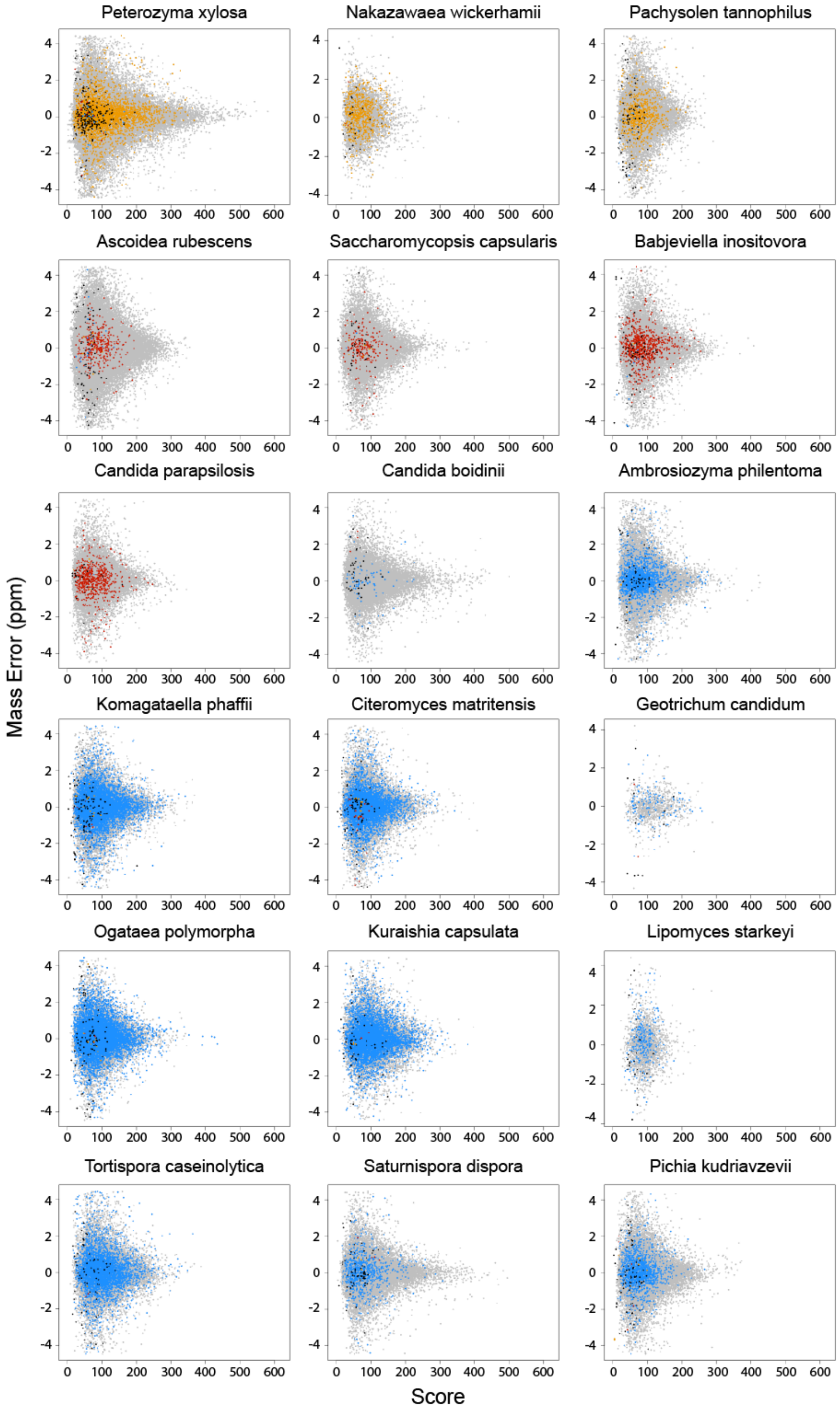

Supplementary Fig. 4. MaxQuant scatterplots showing calculated mass error (parts per million) versus score for all identified MS/MS spectra. All identified peptides not containing CUG-encoded amino acids are colored gray. Peptides containing CUG-translated amino acids are differentially colored: red, CUG-Ser; yellow, CUG-Ala; blue, CUG-Leu; black, CUG matching any amino acid other than Ser, Ala or Leu.

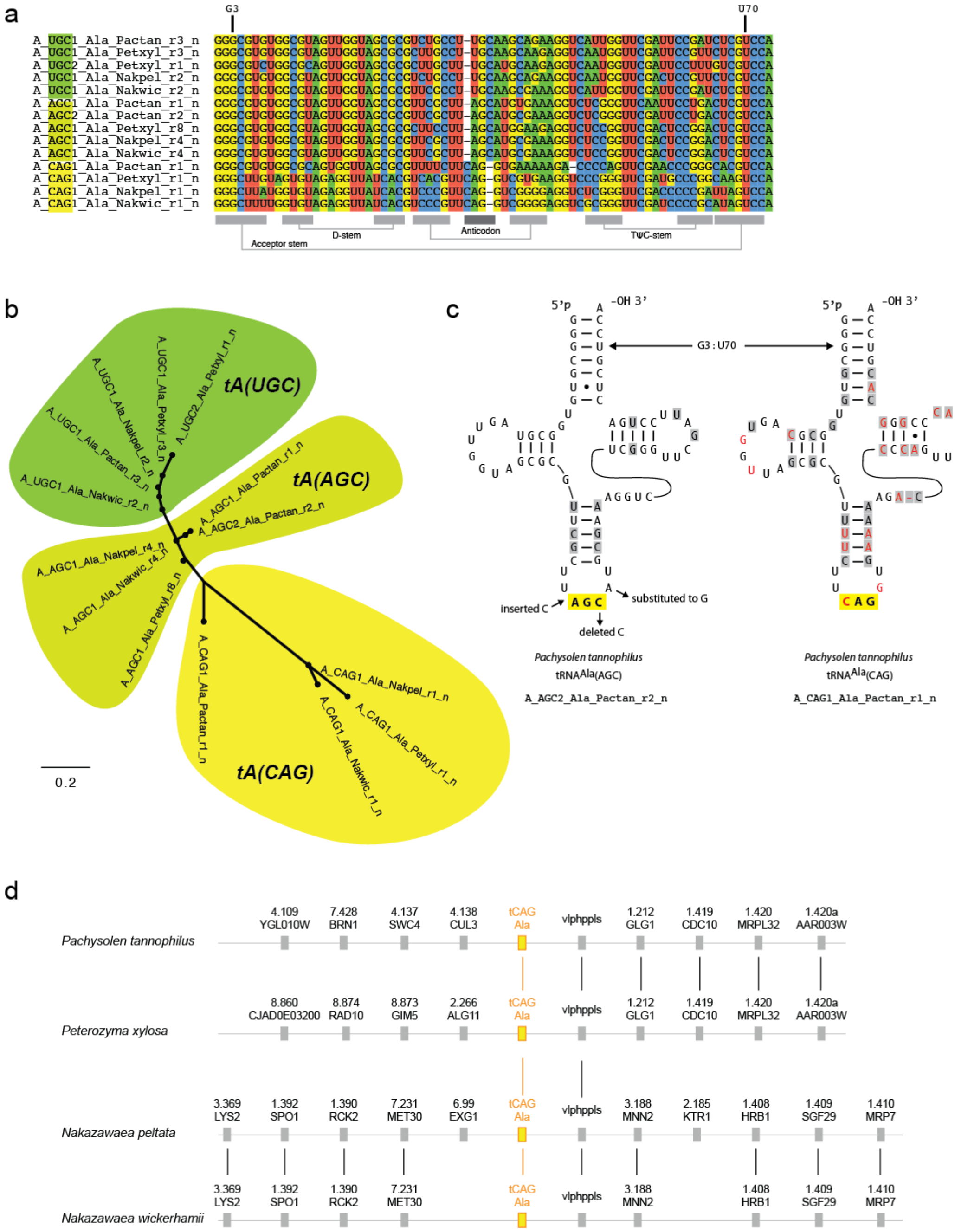

Supplementary Fig. 5. Origin of the tA^{CAG} gene in the Ala clade. **(a)** Alignment of all $tRNA^{Ala}$ sequences from species in the Ala clade. Sequences were aligned manually. **(b)** Unrooted phylogenetic tree constructed from the alignment by maximum likelihood. Some species contain multiple genes coding for identical tRNA sequences, as shown by 'r2', 'r3' (etc.) in the tRNA name to indicate the number of repeats. **(c)** Cloverleaf structures of *P. tannophilus* $tRNA^{Ala}$(CAG) and one of its two types of $tRNA^{Ala}$(AGC) molecules. Gray backgrounds indicate positions that vary among species of the Ala clade, for each tRNA. Red letters indicate positions that differ between the two sequences. The G_3:U_{70} basepair that is a hallmark of alanine tRNAs[1] is indicated. **(d)** Synteny relationship among the four Ala clade species at the tA^{CAG} locus. Genes are named according to their *S. cerevisiae* ortholog where possible, and numbers indicate their locations in the pre-WGD Ancestral genome reconstruction[2]. The name "vlphppls" is used for a gene with no ortholog in baker's yeast, which contains this amino acid sequence motif.

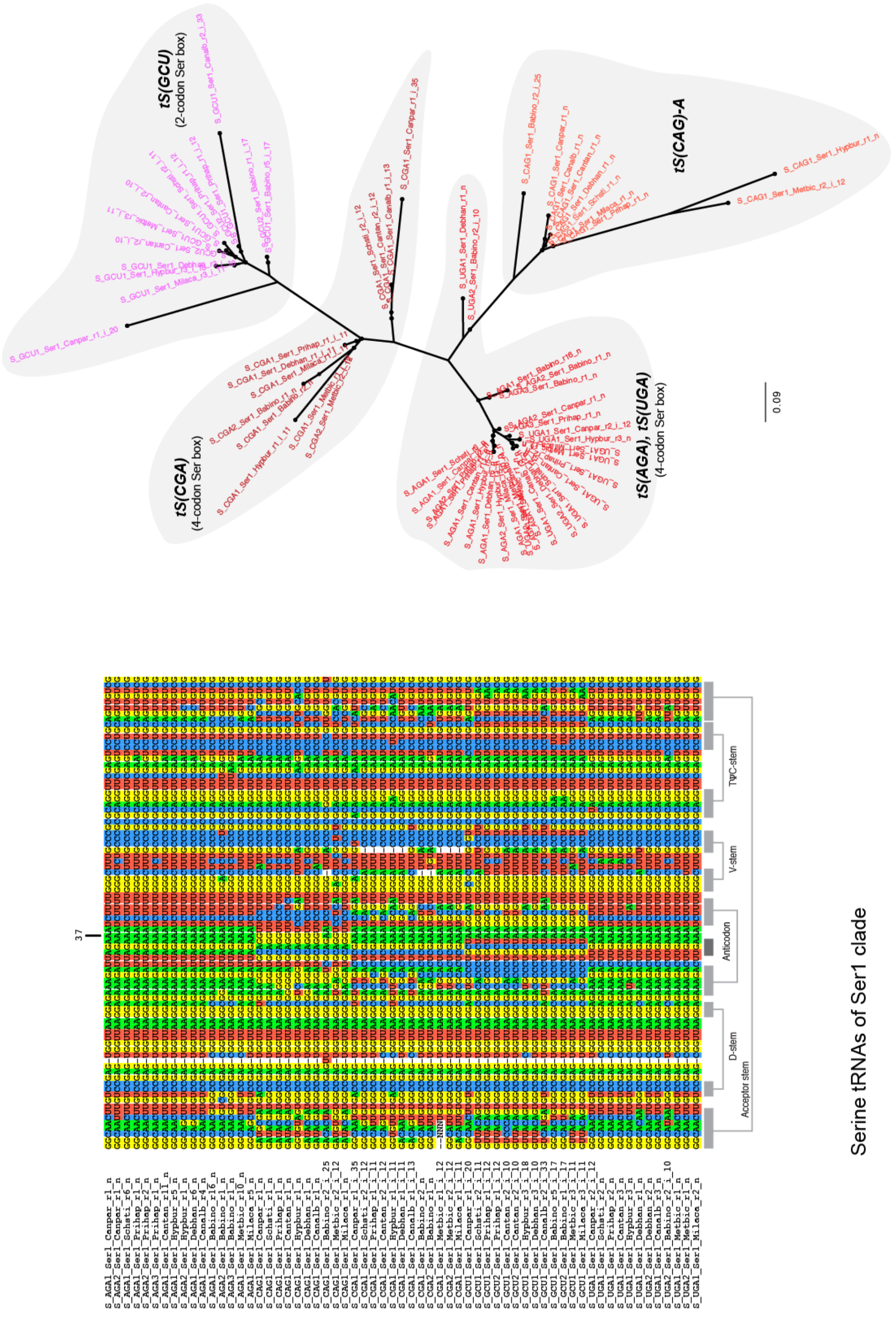

Supplementary Fig. 6. Origin of the tS^{CAG}-*A* gene of the Ser1 clade. A multiple alignment and a phylogenetic tree of all tRNA^{Ser} genes from Ser1 clade species are shown. Position 37 permits low-level misacylation of tRNA^{Ser} by LeuRS when it is G_{37} but not A_{37} (ref. [3]). This position is G_{37} in tRNA^{Ser}(CAG) of most Ser1 clade species, but A_{37} in tRNA^{Ser}(CAG) of *B. inositovora* and *Candida cylindracea* (ref. [3]) as well as in all other tRNA^{Ser} isoacceptors.

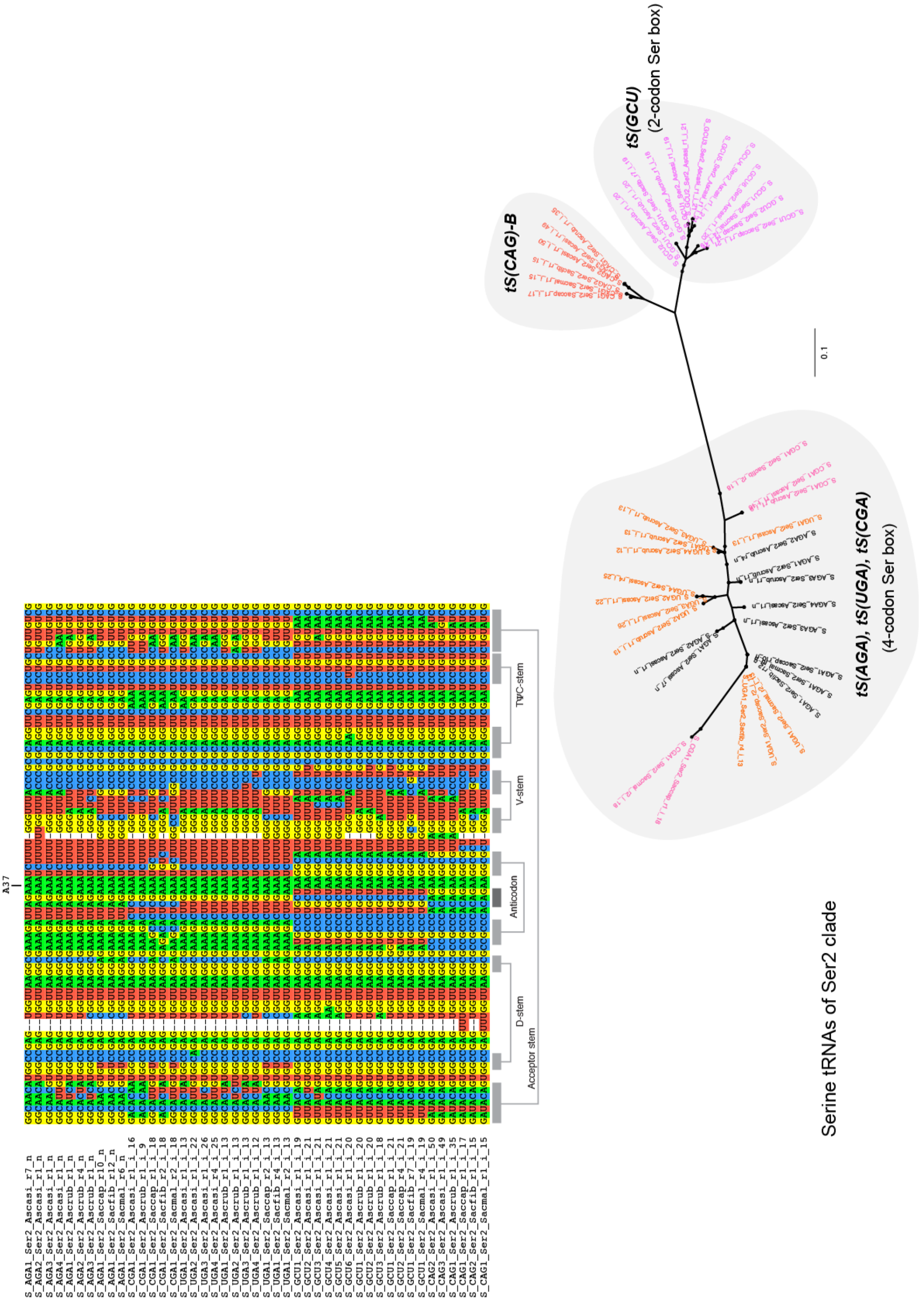

Supplementary Fig. 7. Origin of the tS^{CAG}*-B* gene of the Ser2 clade. A multiple alignment and a phylogenetic tree of all $tRNA^{Ser}$ genes from Ser2 clade species are shown. The A_{37} position that prevents low-level misacylation by LeuRS (ref. [3]) is marked.

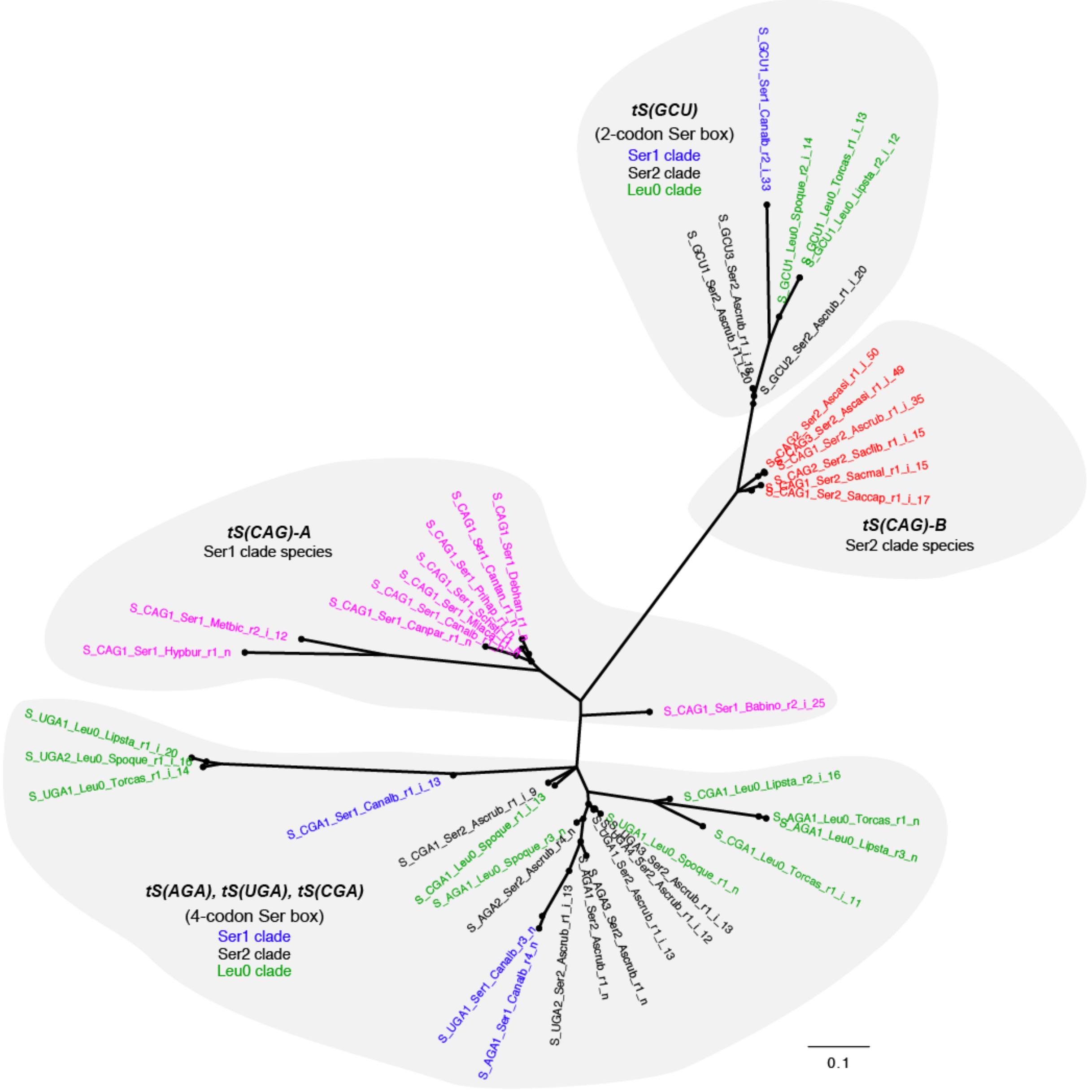

Supplementary Fig. 8. Relationship of tS^{CAG}*-A* from the Ser1 clade, and tS^{CAG}*-B* from the Ser2 clade, to $tRNA^{Ser}$ genes from outgroup species. The outgroups are three species from the Leu0 group (in green), chosen because they have low levels of tRNA gene duplication: *Sporopachydermia quercuum*, *Tortispora caseinolytica*, and *Lipomyces starkeyi*. For reference, all $tRNA^{Ser}$ genes from *Candida albicans* (Ser1 clade) and *Ascoidea rubescens* (Ser2 clade) are also included.

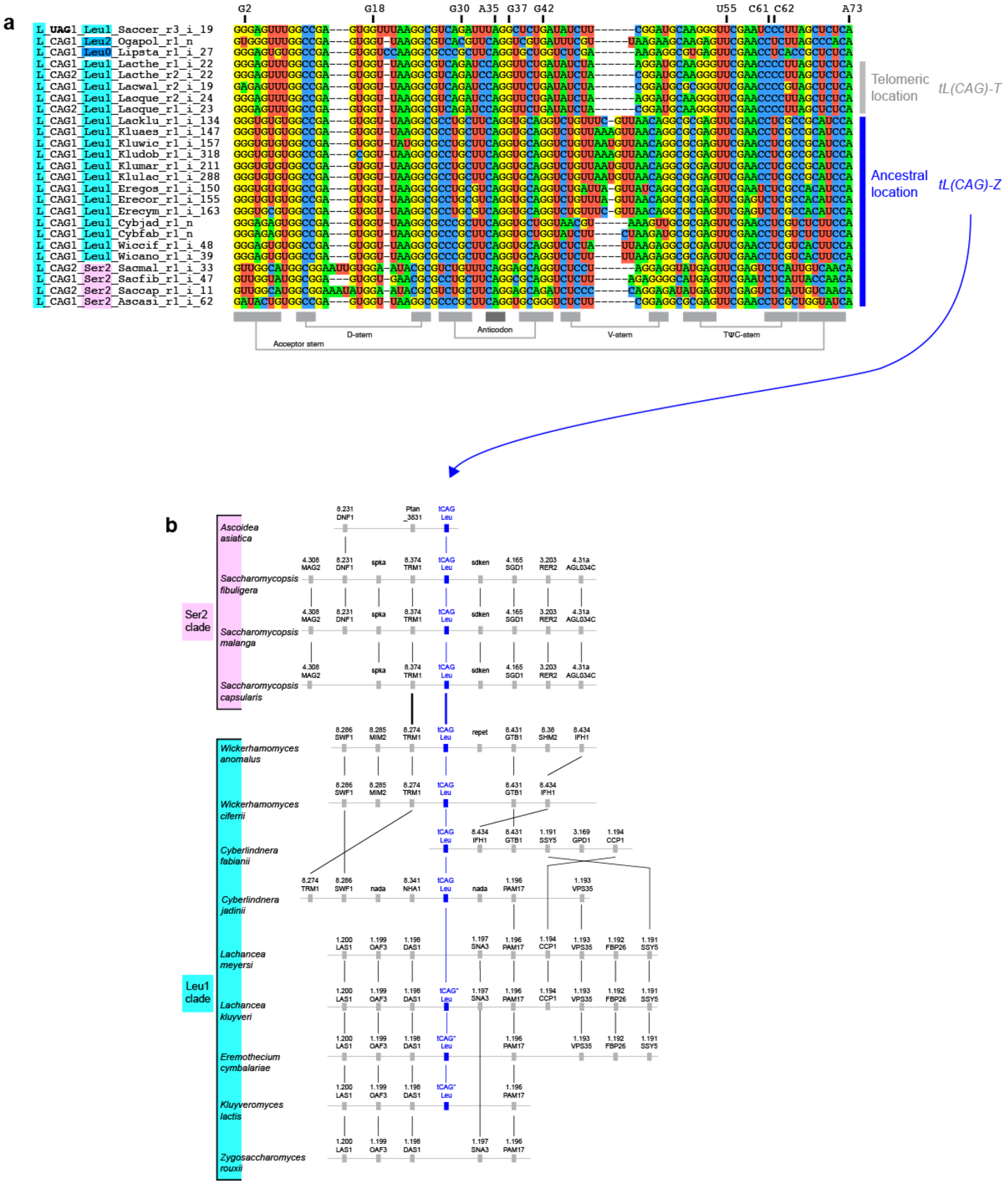

Supplementary Fig. 9 (continues on next page). Orthology of the *tL*^{CAG} genes of the Ser2 and Leu1 clades. **(a)** Alignment of the four *tL*^{CAG} genes identified in the Ser2 clade, with selected *tL*^{CAG} genes from Leu1, Leu0 and Leu2 clades. Sequences were aligned manually. Essential nucleotides in *S. cerevisiae* tRNA^{Leu}(UAG) or other tRNA^{Leu} molecules[1,4] are shown at the top. In particular, the G_{37} and A_{73} positions establish that the Ser2 clade genes code for a tRNA^{Leu}. **(b)** Synteny relationships around *tL*^{CAG}-*Z* genes from Ser2 and Leu1 clades. Genes are named according to their *S. cerevisiae* ortholog where possible, and numbers indicate their locations in the pre-WGD Ancestral genome reconstruction[2]. Asterisks indicate *tL*^{CAG} genes with long introns. *tL*^{CAG} is linked to *TRM1*, which codes for a tRNA-modifying enzyme that makes N2,N2-dimethyl G_{26} and helps tRNAs to fold[5,6].

c

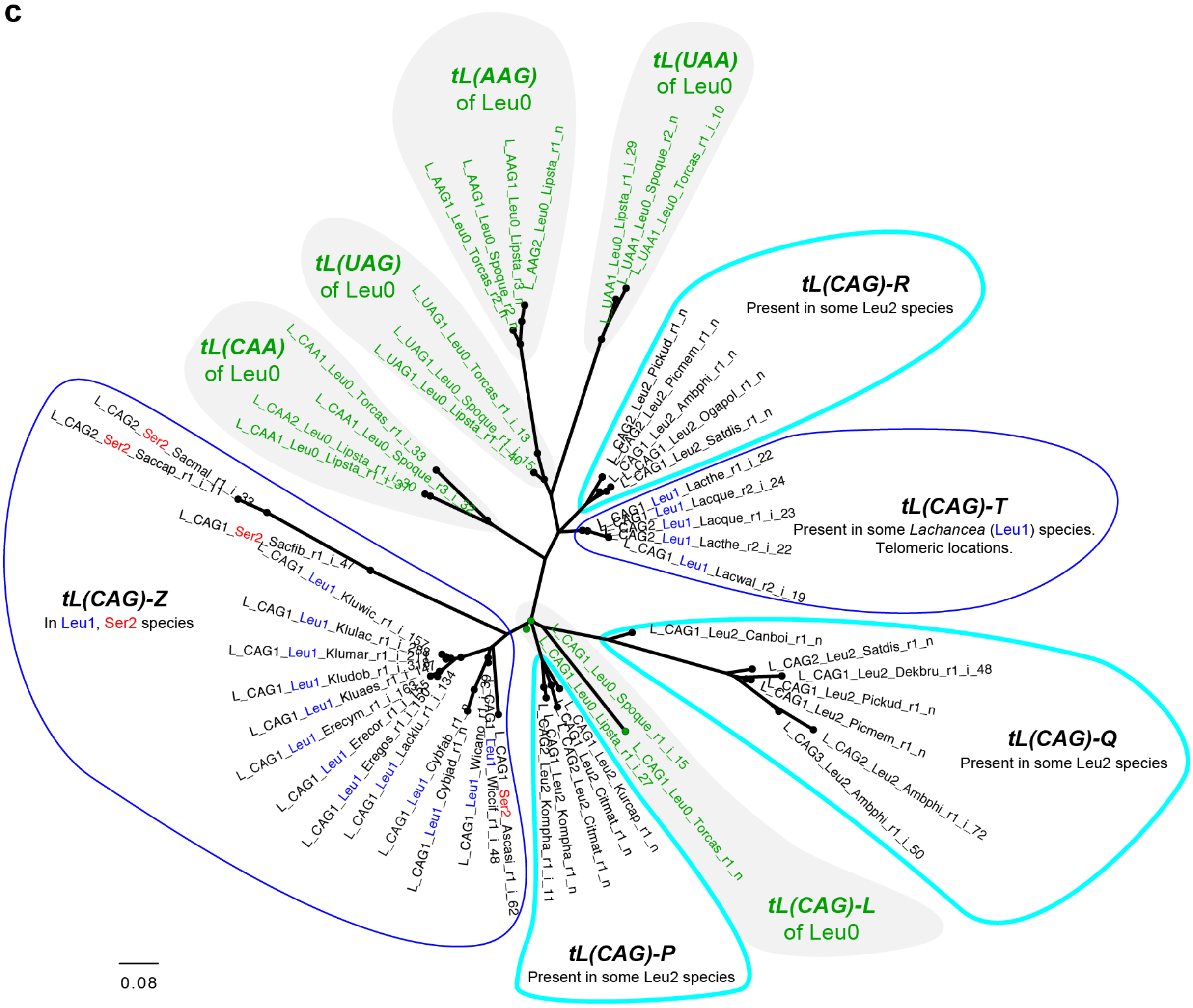

Supplementary Fig. 9 (continued from previous page). (c) Unrooted phylogenetic tree of tL^{CAG} genes from Leu1, Leu2 and Ser2 clades with, as outgroups, other $tRNA^{Leu}$ isoacceptor genes from the Leu0 species *Lipomyces starkeyi*, *Tortispora caseinolytica* and *Sporopachydermia quercuum.* Suffixes -*Z*, -*P*, -*L*, -*Q*, -*T* and -*R* denote different orthogroups of tL^{CAG}.

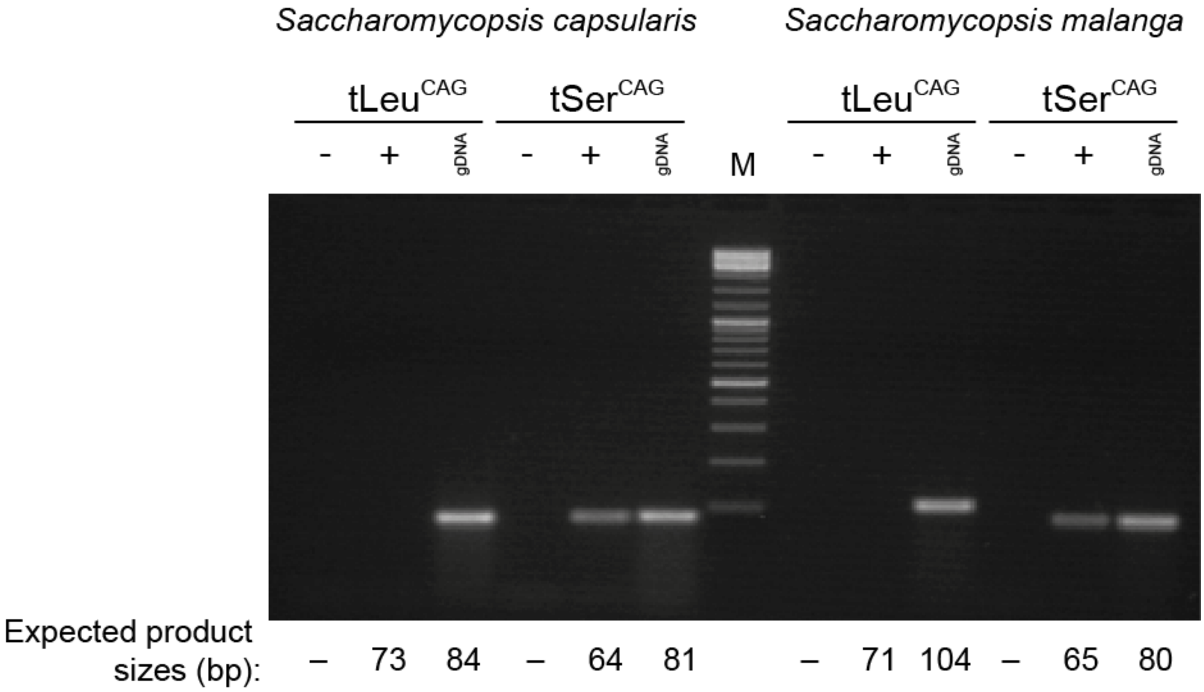

Supplementary Fig. 10. Analysis of expression of tS^{CAG} and tL^{CAG} in *Saccharomycopsis capsularis* and *S. malanga*. Primers specific for tS^{CAG} and tL^{CAG} in each species were used to amplify genomic DNA (gDNA) by PCR, and cDNA by RT-PCR in the presence (+) or absence (-) of reverse transcriptase. The smallest band in the molecular weight ladder (M) is 100 bp. Surprisingly, only 1 of 10 tS^{CAG} cDNAs that we cloned and sequenced from *S. malanga* was spliced, and in *S. capsularis* none of 8 were spliced.

```
Saccharomycopsis_fibuligera     aatcactaacctgtgcctcagatagcacaaaaccgaacagtagaattacagtgcagtttt
Saccharomycopsis_capsularis     cagcataaagacagc----aaagcgcatgcaaaagatgcatagag----ccagataaaca
Saccharomycopsis_malanga        cgttttttaagccc------caagacaatgcga----tatctagga----cccgaaact-a
                                       **             *    *    *        ***         *

Saccharomycopsis_fibuligera     ccagccgctgcttgttacggcggctgct-------tgttacggccgccg-cttgtgacac
Saccharomycopsis_capsularis     ccacatcctgattgaagacgggtaaccctg-tcatgcgcgccttcccgcttctgcgctac
Saccharomycopsis_malanga        cgttacgcctattgttggagtgcacgatgcgcgggtgtcactttcaatgtgttgcaccac
                                *      *   ***     * *                  *   *       **    **

Saccharomycopsis_fibuligera     aaaaac---aca----cagt--gagagagctgcacactagcagacaaggtcaaaacttga
Saccharomycopsis_capsularis     aacatgaacaagaggagagttcaaagacgctcgaggacaagctatacagccaaagcttgt
Saccharomycopsis_malanga        aacagctaaacaaggtcagaacaagctgaaaagaatagcacatgccaaaacaaggccttg
                                ** *     *       **    *         *                ***  * *

Saccharomycopsis_fibuligera     taaacaa--------------accctgaggaaaaacaagccaaacagcttatcaggagca
Saccharomycopsis_capsularis     caaa-----aaaacgccacaaaccccagaacaaagga-agcatttaacaacagaaacaaa
Saccharomycopsis_malanga        ctaacaagctatatgccttagaattcaaggcaaacccagaataaaagtattcaagcagta
                                  **                 *         ***           *       *     *

Saccharomycopsis_fibuligera     GTTGGTATGGCGGA---GTGGTGAACGCGCCTGCTTCAGGttaaagctgcttatacacct
Saccharomycopsis_capsularis     GTTGGCATGGCGGAAATATGGAATACGCGTCTGCTTCAGGtctacaggaca---------
Saccharomycopsis_malanga        GTTGGCATGGCGGAATTGTGGAATACGCGTCTGTTTCAGGgcctaaggttggtttctacc
                                ***** ********    ***   ***** *** ******

Saccharomycopsis_fibuligera     tcacttcagggtgaggcagcgtgatctCGCAGGTCTCTTAGAGGGGCATGAGTTCGAATC
Saccharomycopsis_capsularis     ---------------------------AGCAGATCTCCCCAGGAGATATGAGTTCGAGTC
Saccharomycopsis_malanga        aatc--------------aaaaggactAGCAGGTCTCCT-AGGAGGTATGAGTTCGAGTC
                                                            **** ****     * *  ********** **

Saccharomycopsis_fibuligera     TCATTACCAACAccttctttttttaatctttggttgttataatcagtgctttcctatatg
Saccharomycopsis_capsularis     TCATTGTCAACAtgttcttttttttttaccagttgaagtactggtacttcttttcttag--
Saccharomycopsis_malanga        TCATTGTCAACActttttttttttttatttttttaccaattaacttactgttaaatatgg--
                                *****  *****  ** *******             *      * *  *

Saccharomycopsis_fibuligera     tcatatctccttttggctatgaacataggtttgacgccctcacaaacagca--acaaacc
Saccharomycopsis_capsularis     ---tagacctaatggcctgttt--------gctattcccagtttctaaaca--ggtactt
Saccharomycopsis_malanga        ---taacaatcttttaccattt--------ttatttcctacactgaaggcatcgcatatt
                                   **       *   *  *                **           **

Saccharomycopsis_fibuligera     aat-atattttttttttcttt---ttatttttctccaaacgaaattttggatgacaaaaag
Saccharomycopsis_capsularis     at--cgatgtttacggcttccaaacgggtgtatgccaaatacatctgcaaggtaaatttt
Saccharomycopsis_malanga        ataacgattttctctgagattaagcgccgatc-------------ttccatgccttcttt
                                *     ** **                   *              *   * *

Saccharomycopsis_fibuligera     taccaattaagac----gaagctgaaaatttgcttccatccactggcttaaatactttga
Saccharomycopsis_capsularis     tgcaagtcgatctaccatatgggtaaggtagccctggtttgccataataca---------
Saccharomycopsis_malanga        agtccatcactct----gtacagtaaacactctgttgatccacatggaagctcacttt--
                                      *                 **        *   *   *

Saccharomycopsis_fibuligera     ctatggctcaacttcagcccact----gtatattgcaatatatcaacttccctcttttgt
Saccharomycopsis_capsularis     -----------tcaagccgaaatatttgcataaacactctcagtaagaatcgtctggtgt
Saccharomycopsis_malanga        ----taggcccctttgggcaaatatttgagcatggcatt------actatagtcttgttt
                                                    * *    *   *             *      ***  * *
```

Supplementary Fig. 11. Multiple sequence alignment of tL^{CAG}-*Z* genes and their flanking regions from three *Saccharomycopsis* species. Grey highlighting shows the tRNA genes, with exons in uppercase. Sequence alignment was made by Clustal Omega with manual editing.

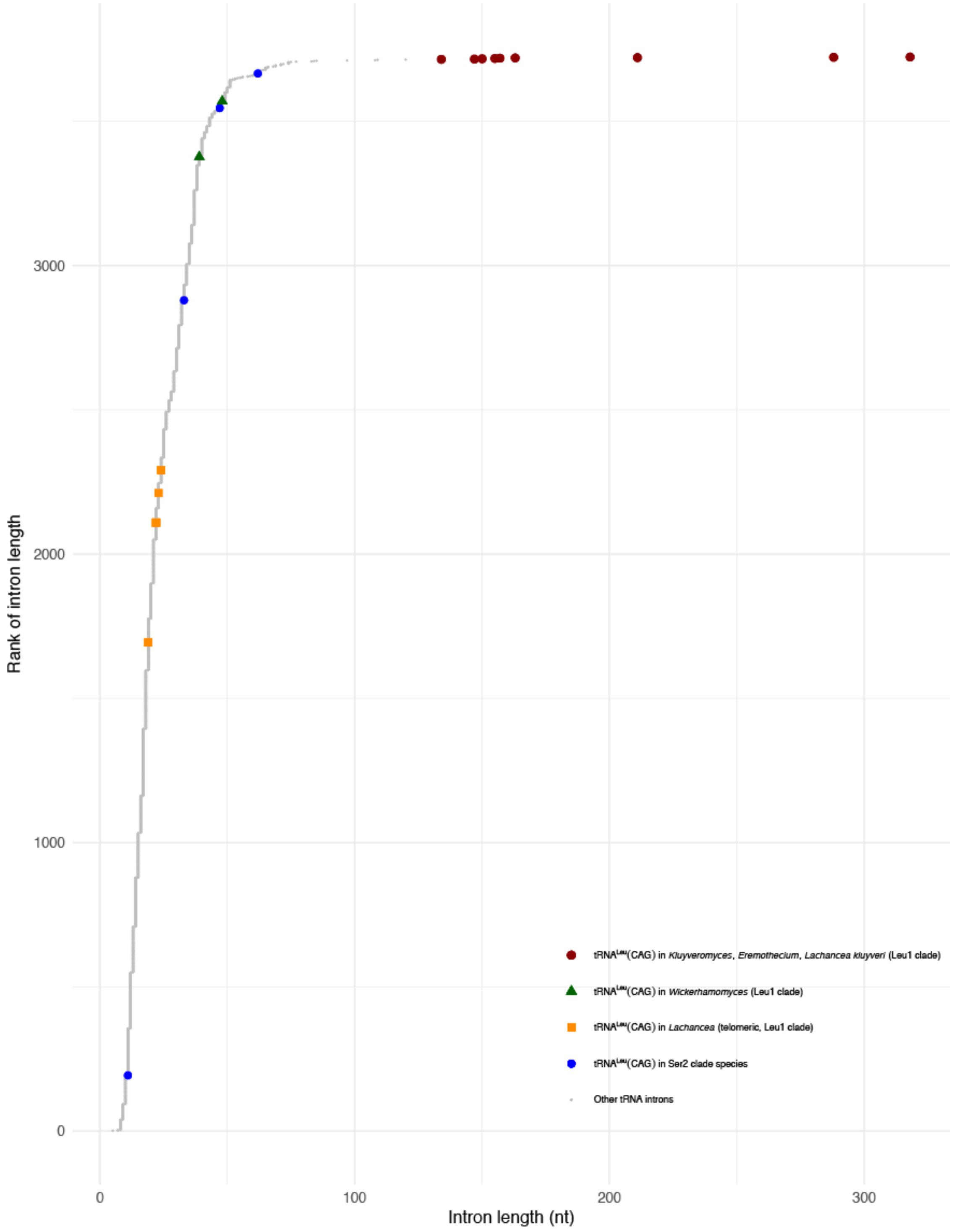

Supplementary Fig. 12. Cumulative distribution of intron lengths in budding yeast tRNA genes. The tRNAomes of 85 Saccharomycotina species were annotated using tRNAscan-SE and manual searches, resulting in 3,723 predicted tRNA introns which were then ranked by length. The lengths of introns in tL^{CAG} in different clades are highlighted.

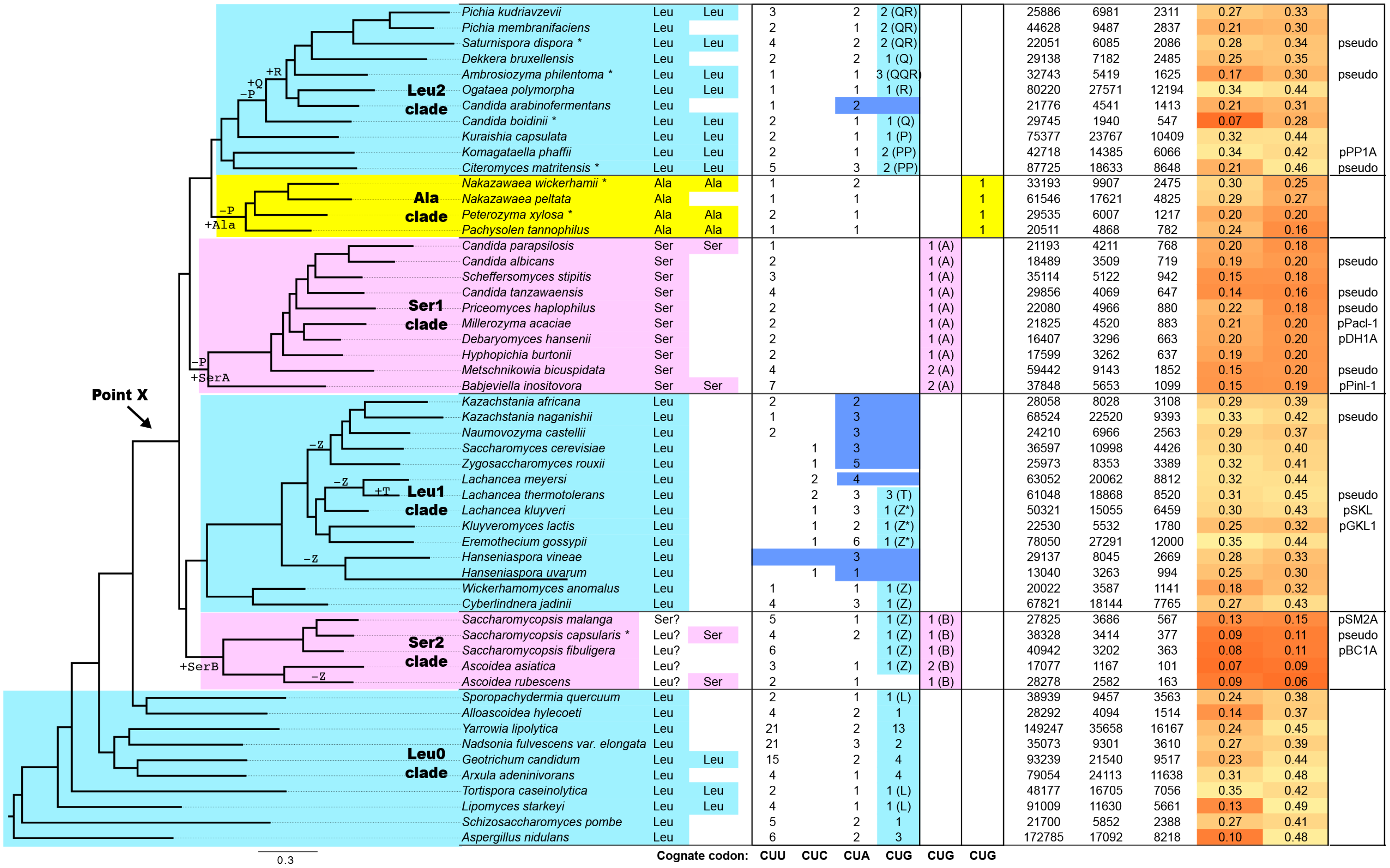

Species	BLAST	Mass Spec	Numbers of tRNA genes: tRNA Leu AAG	tRNA Leu GAG	tRNA Leu UAG	tRNA Leu CAG	tRNA Ser CAG	tRNA Ala CAG	CUG codons in ORFs (a)	CUG codons in BUSCO genes (b)	CUG codons in HSPs (c)	Ratio b/a	Ratio c/b	VLE Content
Pichia kudriavzevii	Leu	Leu	3		2	2 (QR)			25886	6981	2311	0.27	0.33	
Pichia membranifaciens	Leu		2		1	2 (QR)			44628	9487	2837	0.21	0.30	
Saturnispora dispora *	Leu	Leu	4		2	2 (QR)			22051	6085	2086	0.28	0.34	pseudo
Dekkera bruxellensis	Leu		2		2	1 (Q)			29138	7182	2485	0.25	0.35	
Ambrosiozyma philentoma *	Leu	Leu	1		1	3 (QQR)			32743	5419	1625	0.17	0.30	pseudo
Ogataea polymorpha	Leu	Leu	1		1	1 (R)			80220	27571	12194	0.34	0.44	
Candida arabinofermentans	Leu		1		2				21776	4541	1413	0.21	0.31	
Candida boidinii *	Leu	Leu	2		1	1 (Q)			29745	1940	547	0.07	0.28	
Kuraishia capsulata	Leu	Leu	2		1	1 (P)			75377	23767	10409	0.32	0.44	
Komagataella phaffii	Leu	Leu	2		1	2 (PP)			42718	14385	6066	0.34	0.42	pPP1A
Citeromyces matritensis *	Leu	Leu	5		3	2 (PP)			87725	18633	8648	0.21	0.46	pseudo
Nakazawaea wickerhamii *	Ala	Ala	1		2			1	33193	9907	2475	0.30	0.25	
Nakazawaea peltata	Ala		1		1			1	61546	17621	4825	0.29	0.27	
Peterozyma xylosa *	Ala	Ala	2		1			1	29535	6007	1217	0.20	0.20	
Pachysolen tannophilus	Ala	Ala	1		1			1	20511	4868	782	0.24	0.16	
Candida parapsilosis	Ser	Ser	1				1 (A)		21193	4211	768	0.20	0.18	
Candida albicans	Ser		2				1 (A)		18489	3509	719	0.19	0.20	pseudo
Scheffersomyces stipitis	Ser		3				1 (A)		35114	5122	942	0.15	0.18	
Candida tanzawaensis	Ser		4				1 (A)		29856	4069	647	0.14	0.16	pseudo
Priceomyces haplophilus	Ser		2				1 (A)		22080	4966	880	0.22	0.18	pseudo
Millerozyma acaciae	Ser		2				1 (A)		21825	4520	883	0.21	0.20	pPacl-1
Debaryomyces hansenii	Ser		2				1 (A)		16407	3296	663	0.20	0.20	pDH1A
Hyphopichia burtonii	Ser		2				1 (A)		17599	3262	637	0.19	0.20	
Metschnikowia bicuspidata	Ser		4				2 (A)		59442	9143	1852	0.15	0.20	pseudo
Babjeviella inositovora	Ser	Ser	7				2 (A)		37848	5653	1099	0.15	0.19	pPinl-1
Kazachstania africana	Leu		2		2				28058	8028	3108	0.29	0.39	
Kazachstania naganishii	Leu		1		3				68524	22520	9393	0.33	0.42	pseudo
Naumovozyma castellii	Leu		2		3				24210	6966	2563	0.29	0.37	
Saccharomyces cerevisiae	Leu			1	3				36597	10998	4426	0.30	0.40	
Zygosaccharomyces rouxii	Leu			1	5				25973	8353	3389	0.32	0.41	
Lachancea meyersi	Leu			2	4				63052	20062	8812	0.32	0.44	
Lachancea thermotolerans	Leu			2	3	3 (T)			61048	18868	8520	0.31	0.45	pseudo
Lachancea kluyveri	Leu			1	3	1 (Z*)			50321	15055	6459	0.30	0.43	pSKL
Kluyveromyces lactis	Leu			1	2	1 (Z*)			22530	5532	1780	0.25	0.32	pGKL1
Eremothecium gossypii	Leu			1	6	1 (Z*)			78050	27291	12000	0.35	0.44	
Hanseniaspora vineae	Leu				3				29137	8045	2669	0.28	0.33	
Hanseniaspora uvarum	Leu			1	1				13040	3263	994	0.25	0.30	
Wickerhamomyces anomalus	Leu		1		1	1 (Z)			20022	3587	1141	0.18	0.32	
Cyberlindnera jadinii	Leu		4		3	1 (Z)			67821	18144	7765	0.27	0.43	
Saccharomycopsis malanga	Ser?		5		1	1 (Z)	1 (B)		27825	3686	567	0.13	0.15	pSM2A
Saccharomycopsis capsularis *	Leu?	Ser	4		2	1 (Z)	1 (B)		38328	3414	377	0.09	0.11	pseudo
Saccharomycopsis fibuligera	Leu?		6			1 (Z)	1 (B)		40942	3202	363	0.08	0.11	pBC1A
Ascoidea asiatica	Leu?		3		1	1 (Z)	2 (B)		17077	1167	101	0.07	0.09	
Ascoidea rubescens	Leu?	Ser	2		1		1 (B)		28278	2582	163	0.09	0.06	
Sporopachydermia quercuum	Leu		2		1	1 (L)			38939	9457	3563	0.24	0.38	
Alloascoidea hylecoeti	Leu		4		2	1			28292	4094	1514	0.14	0.37	
Yarrowia lipolytica	Leu		21		2	13			149247	35658	16167	0.24	0.45	
Nadsonia fulvescens var. elongata	Leu		21		3	2			35073	9301	3610	0.27	0.39	
Geotrichum candidum	Leu	Leu	15		2	4			93239	21540	9517	0.23	0.44	
Arxula adeninivorans	Leu		4		1	4			79054	24113	11638	0.31	0.48	
Tortispora caseinolytica	Leu	Leu	2		1	1 (L)			48177	16705	7056	0.35	0.42	
Lipomyces starkeyi	Leu	Leu	4		1	1 (L)			91009	11630	5661	0.13	0.49	
Schizosaccharomyces pombe	Leu		5		2	1			21700	5852	2388	0.27	0.41	
Aspergillus nidulans	Leu		6		2	3			172785	17092	8218	0.10	0.48	
		Cognate codon:	CUU	CUC	CUA	CUG	CUG	CUG						

Supplementary Fig. 13. Details of tRNA gene content and CUG codon content in 52 yeast species. The number of tRNA genes in each genome is shown for tRNAs capable of reading CUN codons. Dark blue boxes indicate the wobbling predicted in the four groups of species that have no $tRNA^{Leu}$(CAG) but translate CUG as Leu. For tRNA genes, letters in parentheses indicate membership of orthogroups (clades of orthologous tRNA genes), and + and – symbols on the tree show inferred points of gain or loss of orthogroups members. Columns a-c show the numbers of CUG codons present in all ORFs in the genome (a), in genes that have significant BLASTP hits to the BUSCO database[7] of conserved Ascomycota proteins (b), and in the regions of these genes that BLASTP aligned to BUSCO proteins (c). The ratios among these numbers are shown. Red shading indicates under-representation of CUG codons in conserved genes (low b/a ratio), and under-representation of CUG codons in conserved regions within genes (low c/b ratio). The VLE Content column shows species that harbor cytoplasmic linear DNA plasmids (named) similar to known killer plasmids, or whose genome contains pseudogene remnants of this type of plasmid (Supplementary Table 3). Other details are as in Figure 2.

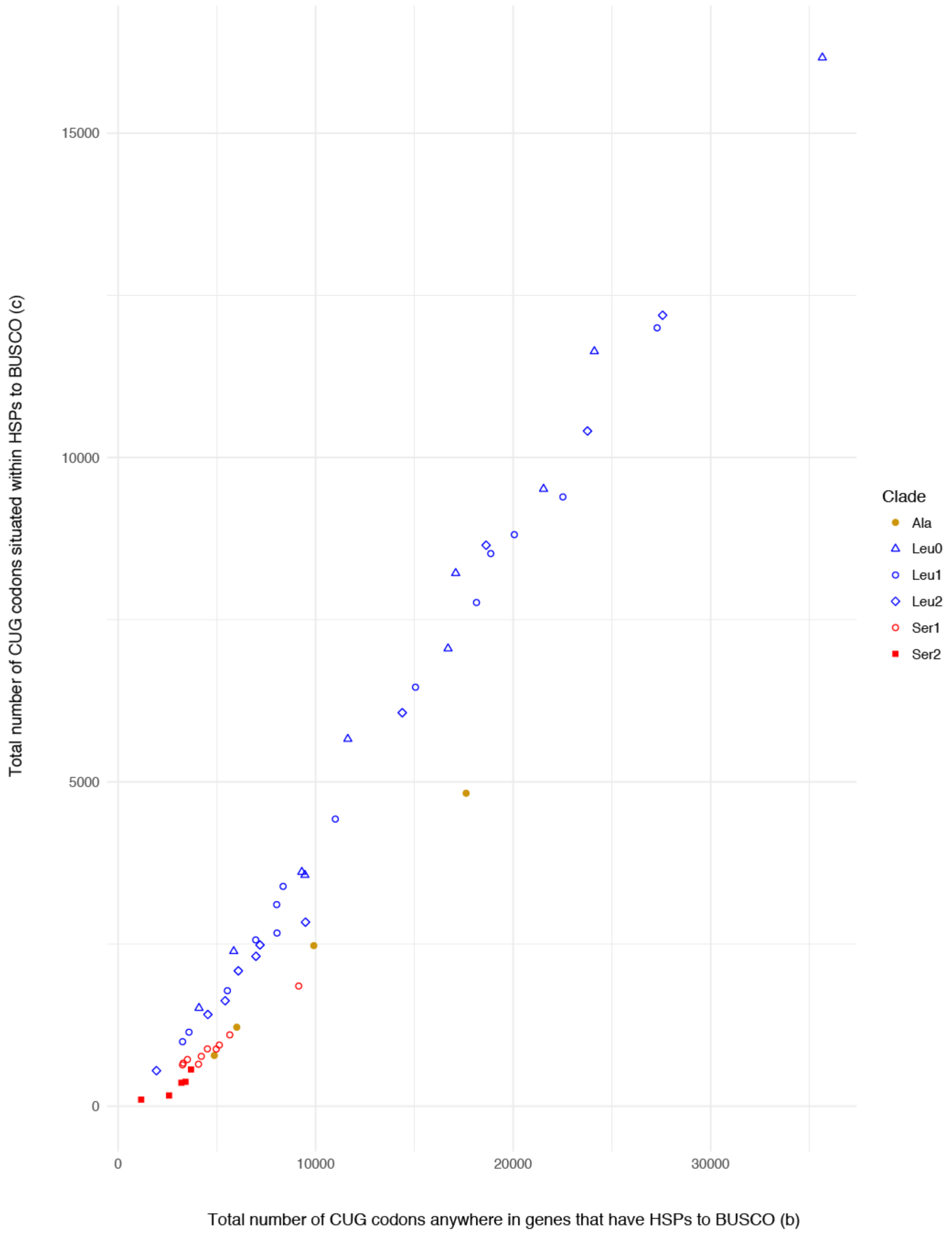

Supplementary Fig. 14. Scatterplot comparing, for yeast species in each clade, the numbers of CUG codons in conserved and non-conserved regions of genes. The predicted genes from each species were compared by BLASTP to the BUSCO database[7] of conserved Ascomycete proteins. For genes that had BLASTP hits with $E < 1\text{e-}10$ to BUSCO, the numbers of CUG codons in the whole gene, and in the region of the gene that formed the BLAST HSP to the BUSCO gene, were calculated. These numbers were then summed for each species. Each point corresponds to one analyzed species, with symbols corresponding to clades. Species in the Ala, Ser1 and Ser2 clades are seen to have fewer CUG codons in total than in the Leu clades, and disproportionately fewer in conserved (HSP-forming) regions of genes. The X and Y axes correspond to columns b and c, respectively, of Supplementary Figure 13.

Supplementary Table 1. CUG site translations with b- and/or y-ion support in MaxQuant analysis of peptide mass fingerprinting data.

		Amino acid corresponding to CUG site																			Total number of CUG sites	Proportion[a]
**Clade**	**Species**	**F**	**L**	**S**	**Y**	**C**	**W**	**P**	**H**	**Q**	**R**	**M**	**T**	**N**	**K**	**V**	**A**	**D**	**E**	**G**		
Leu2	*Ambrosiozyma philentoma*	0	426	0	0	0	0	1	0	0	0	1	0	0	0	0	0	0	0	1	429	0.993
Leu2	*Candida boidinii*	1	32	0	0	0	0	0	0	0	0	0	1	1	1	1	0	0	0	0	37	0.865
Leu2	*Citeromyces matritensis*	0	1547	2	1	0	1	2	0	0	0	0	2	0	0	1	0	1	0	0	1557	0.994
Leu2	*Komagataella phaffii*	0	1645	0	0	0	0	0	2	1	0	0	1	2	1	1	1	0	0	0	1654	0.995
Leu2	*Kuraishia capsulata*	0	1552	0	0	1	1	0	0	0	1	0	0	0	0	1	1	2	1	0	1560	0.995
Leu2	*Ogataea polymorpha*	1	2808	0	0	0	0	1	0	1	0	0	0	0	0	1	2	1	0	0	2815	0.998
Leu2	*Pichia kudriavzevii*	0	430	0	3	1	2	0	0	1	0	0	0	1	0	2	3	0	0	1	444	0.968
Leu2	*Saturnispora dispora*	0	298	0	2	0	0	1	0	0	1	0	1	0	0	0	2	0	0	2	307	0.971
Ala	*Nakazawaea wickerhamii*	0	1	0	0	0	0	0	0	1	0	0	0	0	0	0	134	0	1	0	137	0.978
Ala	*Pachysolen tannophilus*	1	3	0	0	0	0	0	0	2	1	0	1	2	0	1	243	1	1	0	256	0.949
Ala	*Peterozyma xylosa*	1	4	3	0	2	1	1	1	1	0	2	3	1	0	0	472	0	1	4	497	0.950
Ser1	*Babjeviella inositovora*	0	2	242	0	0	0	0	0	2	0	0	0	0	0	0	0	1	0	0	247	0.980
Ser1	*Candida parapsilosis*	0	1	146	1	0	0	0	0	0	0	0	1	1	0	1	1	0	1	0	153	0.954
Ser2	*Ascoidea rubescens*	0	3	59	0	0	0	0	0	0	0	0	1	1	0	3	3	0	3	1	74	0.797
Ser2	*Saccharomycopsis capsularis*	1	1	78	1	0	0	0	0	1	0	0	0	0	1	0	1	1	0	1	86	0.907
Leu0	*Geotrichum candidum*	0	59	0	0	0	0	0	0	0	0	1	0	0	0	1	0	0	0	0	61	0.967
Leu0	*Lipomyces starkeyi*	0	105	0	0	1	0	0	0	1	1	0	1	1	0	0	0	0	0	0	110	0.955
Leu0	*Tortispora caseinolytica*	1	1373	0	1	1	0	0	0	0	0	0	2	2	0	3	0	2	2	0	1387	0.990

Numbers in each cell refer to numbers of genomic CUG sites with a particular translation. Where multiple spectra were obtained that span the same genomic site, all spectra were required to agree regarding translation of the site.

[a]Proportion of CUG sites with the highlighted translation.

Supplementary Table 2. Summary of alternative topologies (some of which are partially resolved) associated with placements of the Ser1/2, Ala and Leu1/2 clades.

Data matrices	Reference Topology[a]	Topological Constraint Tested	diff *lnL*[b]	AU[c]	SH[d]
54taxa_1237AA_unpartitioned	T1	(((Leu1,Leu2),(Ser1,Ser2), Ala),others)	39722.4	3.00E-66*	0*
	T1	((Leu1,Leu2),others)	36338.0	7.00E-46*	0*
	T1	((Ser1,Ser2),others)	6035.5	1.00E-05*	0*
54taxa_1237AA_partitioned	T1	(((Leu1,Leu2),(Ser1,Ser2), Ala),others)	38889.6	3.00E-72*	0*
	T1	((Leu1,Leu2),others)	35685.2	3.00E-41*	0*
	T1	((Ser1,Ser2),others)	5884.3	1.00E-06*	0*

[a] T1 is the Maximum Likelihood tree.
[b] Log-likelihood difference between T1 and the best fully resolved topology that satisfies the topological constraint.
[c] Approximately Unbiased test[8]. Asterisks indicate significant support for the fully resolved topology over the alternative topology (*i.e.*, for rejecting the alternative topology as an equally likely explanation of the data).
[d] Shimodaira-Hasegawa test[9]. Asterisks indicate significant support for the fully resolved topology over the alternative topology (*i.e.*, for rejecting the alternative topology as an equally likely explanation of the data).

Supplementary Table 3. Virus-Like Elements (VLEs) and their pseudogenes.

(A) Known cytoplasmic linear DNA killer plasmids and accessory plasmids, including unsequenced ones (from Fukuhara[10]).

Plasmid name	Host species	Clade	NCBI accession number	Synonym
pGKL1, 2	*Kluyveromyces lactis*	Leu1	X01095.1	
pSKL	*Lachancea kluyveri*	Leu1	X54850.1	*Saccharomyces kluyveri*
pPP1A, pPP1B	*Komagataella phaffii*	Leu2	A.Y.C. & K.H.W., unpublished	*Pichia pastoris*
pPH1	*Pichia heedi*	Leu2	Not sequenced	
pPK1	*Pichia kluyveri*	Leu2	Not sequenced	
pPinl-1, 2, 3	*Babjeviella inositovora*	Ser1	AJ564102.1	*Pichia inositovora*
pDH1A	*Debaryomyces hansenii*	Ser1	JN624283.1	
pDP1	*Debaryomyces polymorphus*	Ser1	Not sequenced	
pWR1A	*Debaryomyces robertsiae*	Ser1	AJ617332.1	
pPacl-1, 2	*Millerozyma acaciae*	Ser1	AM180622.1	*Pichia acaciae*
pPE1B	*Schwanniomyces etchellsii*	Ser1	AJ278986.2	*Pichia etschellsii*
pScrl-1, 2, 3	*Saccharomycopsis crataegensis*	Ser2	Not sequenced	
pBC1A, B	*Saccharomycopsis fibuligera*	Ser2	Not sequenced	*Botryoascus cladosporoides*
pSM2A	*Saccharomycopsis malanga*	Ser2	Not sequenced	

(B) Genomic pseudogenes of cytoplasmic linear DNA plasmids.

Query plasmid name	Host species of query plasmid	Query plasmid protein	Type of protein from query plasmid	Subject species	Clade of subject species	Subject accession number / chromosome / contig / scaffold	Subject location (bp position)	TBLASTN E-value
pGKL2	*Kluyveromyces lactis*	ORF9	Other	*Kazachstania africana*	Leu1	NC_018946.1	159865..159716	4E-11
pGKL1	*Kluyveromyces lactis*	URFP1	DNA polymerase	*Kluyveromyces lactis*	Leu1	NC_006042.1 (chromosome F)	2602192..2601947	9E-40
pGKL1	*Kluyveromyces lactis*	URFP4	Other	*Kluyveromyces lactis*	Leu1	NC_006040.1 (chromosome D)	1519466..1519323	3E-140
pGKL1	*Kluyveromyces lactis*	URFP2	Chitin-binding protein	*Lachancea meyersii*	Leu1	FJUM01000015.1	87694..90096	0E+00
pSKL	*Lachancea kluyveri*	ORF2	DNA polymerase	*Lachancea meyersii*	Leu1	FJUM01000002.1	785282..785626	8E-21
pSKL	*Lachancea kluyveri*	ORF1	Other	*Lachancea meyersii*	Leu1	FJUM01000002.1	786058..785843	3E-12
pPP1A	*Komagataella phaffii*	ORF2	DNA polymerase	*Ambrosiozyma philentoma*	Leu2	NHAR0000000, NODE_29	155848..156321	8E-30
pWR1A	*Debaryomyces robertsiae*	ORF1	Other	*Ambrosiozyma philentoma*	Leu2	NHAR0000000, NODE_39	48746..47553	2E-17
pPinl-3	*Babjeviella inositovora*	ORF2	DNA polymerase	*Citeromyces matritensis*	Leu2	NHAP00000000, scf7180000043097	21318..20980	1E-12
pSKL	*Lachancea kluyveri*	ORF1	Other	*Citeromyces matritensis*	Leu2	NHAP00000000, scf7180000043097	20231..19881	2E-27
pPP1A	*Komagataella phaffii*	ORF1	Chitin-binding protein	*Saturnispora dispora*	Leu2	NHAL00000000, NODE_2	285627..284350	9E-101
pPac1-2	*Millerozyma_acaciae*	ORF1	Chitin-binding protein	*Babjeviella inositovora*	Ser1	scaffold_37	33..596	3E-75
pPP1A	*Komagataella phaffii*	ORF2	DNA polymerase	*Babjeviella inositovora*	Ser1	scaffold_38	1131..1	3E-166
pPP1A	*Komagataella phaffii*	ORF1	Chitin-binding protein	*Candida albicans*	Ser1	CP017623.1	2964425..2965813	2E-143
pPacl-1	*Millerozyma_acaciae*	ORF2	DNA polymerase	*Candida tanzawaensis*	Ser1	scaffold_4	468162..468512	8E-19
pPacl-2	*Millerozyma_acaciae*	ORF1	Chitin-binding protein	*Debaryomyces hansenii*	Ser1	NC_006048.2 (chromosome F)	1415591..1416967	2E-163
pSKL	*Lachancea kluyveri*	ORF2	DNA polymerase	*Debaryomyces hansenii*	Ser1	NC_006044.2 (chromosome B)	1006802..1006921	1E-06
pWR1A	*Debaryomyces robertsiae*	ORF5	Other	*Debaryomyces hansenii*	Ser1	NC_006049.2 (chromosome G)	1355356..1354841	3E-61
pPacl-1	*Millerozyma_acaciae*	ORF2	DNA polymerase	*Metschnikowia bicuspidata*	Ser1	scaffold_2	1597435..1598079	1E-26
pWR1A	*Debaryomyces robertsiae*	ORF5	Other	*Millerozyma acaciae*	Ser1	BCKO01000006	316012..316401	2E-09
pPacl-2	*Millerozyma_acaciae*	ORF1	Chitin-binding protein	*Priceomyces haplophilus*	Ser1	BCIF01000001 (scaffold 0)	779320..777917	6E-115
pPacl-2	*Millerozyma_acaciae*	ORF1	Chitin-binding protein	*Saccharomycopsis capsularis*	Ser2	NHAM00000000, flattened_line_108	63884..63495	8E-34
pPP1A	*Komagataella phaffii*	ORF2	DNA polymerase	*Saccharomycopsis capsularis*	Ser2	NHAM00000000, flattened_line_98	35473..34415	1E-73
pPE1B	*Pichia etchellsii*	ORF4	Other	*Saccharomycopsis capsularis*	Ser2	NHAM00000000, flattened_line_88	33220..33369	9E-11
pSKL	*Lachancea kluyveri*	ORF6	RNA polymerase	*Saccharomycopsis capsularis*	Ser2	NHAM00000000, flattened_line_169	13928..14230	3E-23
pPinl-3	*Babjeviella inositovora*	ORF3	Chitin-binding protein	*Saccharomycopsis fibuligera*	Ser2	CP012825.1	2633570..2632500	8E-42
pPinl-3	*Babjeviella inositovora*	ORF3	Chitin-binding protein	*Saccharomycopsis malanga*	Ser2	BCGJ01000007.1	158965..157898	4E-34
pPinl-3	*Babjeviella inositovora*	ORF2	DNA polymerase	*Saccharomycopsis malanga*	Ser2	BCGJ01000005.1	1620614..1620456	1E-17
pGKL2	*Kluyveromyces lactis*	ORF3	Other	*Saccharomycopsis malanga*	Ser2	BCGJ01000001.1	2557807..2557661	1E-23
pPE1B	*Pichia etchellsii*	ORF6	RNA polymerase	*Saccharomycopsis malanga*	Ser2	BCGJ01000005.1	352248..352051	2E-05

Supplementary Table 4. Details of LC-MS/MS experiments.

Clade	Species	MS scans	MSMS scans	PEAKS peptides	Unique PEAKS peptides	Predicted ORFs hit by PEAKS peptides	CUG codons spanned by PEAKS peptides	MaxQuant peptides @ 1% FDR	Unique MaxQuant peptides	MaxQuant peptides that span a CUG codon with b- or y- ion support
Leu2	*Ambrosiozyma philentoma*	11912	74175	55210	35214	2091	270	40836	14168	689
Leu2	*Candida boidinii*	19324	99827	44145	29379	1749	17	24085	8485	68
Leu2	*Citeromyces matritensis*	12218	75616	59406	34268	1786	1062	41739	13335	2070
Leu2	*Komagataella phaffii*	12173	75290	60348	34407	1953	1115	44153	13409	2295
Leu2	*Kuraishia capsulata*	13047	52908	42944	24706	1253	1094	25322	7327	1827
Leu2	*Ogataea polymorpha*	12239	74733	58587	33006	1811	1891	36139	10933	3280
Leu2	*Pichia kudriavzevii*	12060	73206	57082	32560	1780	250	39963	11354	604
Leu2	*Saturnispora dispora*	19769	103565	45263	28796	1585	176	28120	9091	433
Ala	*Nakazawaea wickerhamii*	9861	52454	22041	16503	709	103	7434	2514	285
Ala	*Pachysolen tannophilus*	12992	67984	48944	35717	2017	160	29750	12050	428
Ala	*Peterozyma xylosa*	42399	242197	160561	66028	2841	372	115265	17477	764
Ser1	*Babjeviella inositovora*	11963	71485	57868	33334	2072	173	35753	12475	387
Ser1	*Candida parapsilosis*	11413	63163	44874	28019	1719	89	34966	11380	290
Ser2	*Ascoidea rubescens*	46787	228199	124267	38246	2154	43	111051	16785	124
Ser2	*Saccharomycopsis capsularis*	12134	75547	59067	37016	2027	57	39849	14354	158
Leu0	*Geotrichum candidum*	10845	37457	27453	15587	923	217	1224	528	80
Leu0	*Lipomyces starkeyi*	11724	67058	48705	29526	1237	141	2527	1134	146
Leu0	*Tortispora caseinolytica*	12818	51440	42006	24609	1407	918	28532	9377	1586

Supplementary Table 5. Monoisotopic residue masses of amino acids, and the allowable mass ranges used for b/y ion fragment determination.

Amino Acid	Monoisotopic residue mass (Da)	Accepted mass range
Glycine	57.02147	56.96 - 57.08
Alanine	71.03712	70.977 - 71.097
Serine	87.03203	86.97 - 87.09
Proline	97.05277	96.99 - 97.11
Valine	99.06842	99.0 - 99.128
Threonine	101.04768	100.98 - 101.1
Cysteine	103.00919	102.9491 - 103.0691
Leucine	113.08407	113.00407 - 113.16407
Asparagine	114.04293	113.982 - 114.102
Aspartic acid	115.02695	114.966 - 115.086
Glutamine	128.05858	127.99858 - 128.0767
Lysine	128.09497	128.0768 - 128.15497
Glutamic Acid	129.0426	128.9826 - 129.1026
Methionine	131.04049	130.98 - 131.1
Histidine	137.059	136.99891 - 137.11891
Phenylalanine	147.06842	147.008 - 147.128
Arginine	156.10112	156.0411 - 156.1611
Cysteine (carbamidomethylated)	160.03065	159.95 - 160.11
Cysteine (carboxymethylated)	161.01466	160.934 - 161.094
Tyrosine	163.06333	163.003 - 163.123
Tryptophan	186.07932	186.019 - 186.139

Supplementary Table 6. Primers used for RT-PCR and genomic PCR of tRNA-Leu(CAG) and tRNA-Ser(CAG) from *Saccharomycopsis malanga* (Smal) and *Saccharomycopsis capsularis* (Scaps).

Smal_tLeu_F	TGGCATGGCGGAATTGTG
Smal_tLeu_R	AGACTCGAACTCATACCTCC
Smal_tSer_F	TACAGTGGCCGAGTTTGGTTAAG
Smal_tSer_R	CGAACCTGCGAGGGAAATC
Scaps_tLeu_F	CATGGCGGAAATATGGAATACG
Scaps_tLeu_R	GACTCGAACTCATATCTCCTGG
Scaps_tSer_F	CAGTGGCCGAGTTTGGTTAAG
Scaps_tSer_R	TCGAACCTGCGAGGGAAATC

Supplementary Table 7. Sources of genome sequence data used in this study.

Group	Species name	Short name	Strain	Source	Reference
Saccharomycotina	*Alloascoidea hylecoeti*	Allhyl	JCM 7604	NCBI	Unpublished, Genbank Accession IDs: BCKZ01000001-BCKZ01000136
	Ambrosiozyma philentoma	Ambphi	NRRL Y-7523	Y1000+	This study, Genbank Accession ID: NHAR0000000
	Arxula adeninivorans	Arxade	LS3	Genolevures	Kunze G, et al. The complete genome of Blastobotrys (Arxula) adeninivorans LS3 - a yeast of biotechnological interest. Biotechnol Biofuels 7, 66 (2014).
	Ascoidea asiatica	Ascasi	JCM 7603	NCBI	Unpublished, Genbank Accession IDs: BCKQ01000001-BCKQ01000071
	Ascoidea rubescens	Ascrub	NRRL Y-17699	JGI	Riley R, et al. Comparative genomics of biotechnologically important yeasts. Proc. Natl. Acad. Sci. U.S.A. 113, 9882-9887 (2016).
	Babjeviella inositovora	Babino	NRRL Y-12698	JGI	Riley R, et al. Comparative genomics of biotechnologically important yeasts. Proc. Natl. Acad. Sci. U.S.A. 113, 9882-9887 (2016).
	Candida albicans	Canalb	SC5314	Genolevures	Jones T, et al. The diploid genome sequence of Candida albicans. Proc Natl Acad Sci USA 2004,101:7329-7334.
	Candida arabinofermentans	Canara	NRRL YB-2248	JGI	Riley R, et al. Comparative genomics of biotechnologically important yeasts. Proc. Natl. Acad. Sci. U.S.A. 113, 9882-9887 (2016).
	*Candida boidinii**	Canboi	NRRL Y-2332*	Y1000+	This study, Genbank Accession ID: NHAQ00000000
	Candida parapsilosis	Canpar	CDC 317	Genolevures	Butler G, et al. Evolution of pathogenicity and sexual reproduction in eight Candida genomes. Nature 459, 657-662 (2009).
	Candida tanzawaensis	Cantan	NRRL Y-17324	JGI	Riley R, et al. Comparative genomics of biotechnologically important yeasts. Proc. Natl. Acad. Sci. U.S.A. 113, 9882-9887 (2016).
	Citeromyces matritensis	Citmat	NRRL Y-2407	Y1000+	This study, Genbank Accession ID: NHAP00000000
	Cyberlindnera jadinii	Cybjad	NRRL Y-1542	JGI	Riley R, et al. Comparative genomics of biotechnologically important yeasts. Proc. Natl. Acad. Sci. U.S.A. 113, 9882-9887 (2016).
	Debaryomyces hansenii	Debhan	CBS 767	Genolevures	Dujon B, et al. Genome evolution in yeasts. Nature 430, 35-44 (2004).
	Dekkera bruxellensis	Dekbru	CBS 2499	JGI	Piskur J, et al. The genome of wine yeast Dekkera bruxellensis provides a tool to explore its food-

				related properties. Int. J. Food Microbiol. 157, 202-209 (2012).
Eremothecium gossypii	Eregos	ATCC 10895	Genolevures	Dietrich FS, et al. The Ashbya gossypii Genome as a Tool for Mapping the Ancient Saccharomyces cerevisiae Genome. Science. 2004 Apr 9;304(5668):304-7.
Geotrichum candidum	Geocan	CLIB 918	NCBI	Morel G, et al. Differential gene retention as an evolutionary mechanism to generate biodiversity and adaptation in yeasts. Sci Rep 5: 11571 (2015).
Hanseniaspora uvarum	Hanuva	AWRI3580	NCBI	Sternes, P. R., Lee, D., Kutyna, D. R. & Borneman, A. R. Genome Sequences of Three Species of Hanseniaspora Isolated from Spontaneous Wine Fermentations: TABLE 1. Genome Announc. 4, e01287-16 (2016).
Hanseniaspora vineae	Hanvin	T02/19AF	NCBI	Giorello FM, et al. Genome Sequence of the Native Apiculate Wine Yeast Hanseniaspora vineae T02/19AF. Genome Announc 2, (2014).
Hyphopichia burtonii	Hypbur	NRRL Y-1933	JGI	Riley R, et al. Comparative genomics of biotechnologically important yeasts. Proc. Natl. Acad. Sci. U.S.A. 113, 9882-9887 (2016).
Kazachstania africana	Kazafr	CBS 2517	JGI	Gordon JL, et al. Evolutionary erosion of yeast sex chromosomes by mating-type switching accidents. Proc. Natl. Acad. Sci. U.S.A. 108, 20024-20029 (2011).
Kazachstania naganishii	Kaznag	CBS 8797	Genolevures	Gordon JL, et al. Evolutionary erosion of yeast sex chromosomes by mating-type switching accidents. Proc. Natl. Acad. Sci. U.S.A. 108, 20024-20029 (2011).
Kluyveromyces lactis	Klulac	CBS 2359	Genolevures	Dujon B, et al. Genome evolution in yeasts. Nature 430, 35-44 (2004).
Komagataella phaffii	Kompha	GS115	JGI	De Schutter K, et al. Genome sequence of the recombinant protein production host Pichia pastoris. Nat. Biotechnol. 27, 561-566 (2009).
Kuraishia capsulata	Kurcap	CBS 1993	Genolevures	Morales L, et al. Complete DNA sequence of Kuraishia capsulata illustrates novel genomic features among budding yeasts (Saccharomycotina). Genome Biol Evol. 2013;5(12):2524-39.
Lachancea kluyveri	Lacklu	CBS 3082	Genolevures	Genolevures Consortium, et al. Comparative genomics of protoploid Saccharomycetaceae. Genome Res. 2009 Oct;19(10):1696-709.Ê
Lachancea meyersi	Lacmey	CBS 8951	Genolevures	Vakirlis N, et al. Reconstruction of ancestral chromosome architecture and gene repertoire reveals principles of genome evolution in a model yeast genus. Genome Res. 2016 Jul;26(7):918-32. doi: 10.1101/gr.204420.116. Epub 2016 May 31.
Lachancea thermotolerans	Lacthe	CBS 6340	Genolevures	Genolevures Consortium, et al. Comparative genomics of protoploid Saccharomycetaceae. Genome Res. 2009 Oct;19(10):1696-709.
Lipomyces starkeyi	Lipsta	NRRL Y-11557	JGI	Riley R, et al. Comparative genomics of biotechnologically important yeasts. Proc. Natl. Acad. Sci. U.S.A. 113, 9882-9887 (2016).
Metschnikowia bicuspidata	Metbic	NRRL TB-4993	JGI	Riley R, et al. Comparative genomics of biotechnologically important yeasts. Proc. Natl. Acad. Sci. U.S.A. 113, 9882-9887 (2016).

Millerozyma acaciae	Milaca	JCM 10732	NCBI	Unpublished, Genbank Accession IDs: BCKO01000001-BCKO01000010
Nadsonia fulvescens var elongata	Nadful	DSM 6958	JGI	Riley R, et al. Comparative genomics of biotechnologically important yeasts. Proc. Natl. Acad. Sci. U.S.A. 113, 9882-9887 (2016).
Nakazawaea peltata	Nakpel	JCM 9829	NCBI	Unpublished, Genbank Accession IDs: BCGQ01000001-BCGQ01000011
Nakazawaea wickerhamii	Nakwic	NRRL Y-2563	Y1000+	This study, Genbank Accession ID: NHAO00000000
Naumovozyma castellii	Naucas	CBS 4309	Genolevures	Gordon JL, et al. Evolutionary erosion of yeast sex chromosomes by mating-type switching accidents. Proc. Natl. Acad. Sci. U.S.A. 108, 20024-20029 (2011).
Ogataea polymorpha	Ogapol	NCYC 495	JGI	Riley R, et al. Comparative genomics of biotechnologically important yeasts. Proc. Natl. Acad. Sci. U.S.A. 113, 9882-9887 (2016).
Pachysolen tannophilus	Pactan	NRRL Y-2460	JGI	Riley R, et al. Comparative genomics of biotechnologically important yeasts. Proc. Natl. Acad. Sci. U.S.A. 113, 9882-9887 (2016).
Peterozyma xylosa	Petxyl	NRRL Y-12939	Y1000+	This study, Genbank Accession ID: NHAN00000000
Pichia kudriavzevii	Pickud	M12	NCBI	Chan GF, et al. Genome sequence of Pichia kudriavzevii M12, a potential producer of bioethanol and phytase. Eukaryotic Cell 11, 1300-1301 (2012).
Pichia membranifaciens	Picmem	CBS 107	JGI	Riley R, et al. Comparative genomics of biotechnologically important yeasts. Proc. Natl. Acad. Sci. U.S.A. 113, 9882-9887 (2016).
Priceomyces haplophilus	Prihap	JCM 1635	NCBI	Unpublished, Genbank Accession IDs: BCIF01000001-BCIF01000009
Saccharomyces cerevisiae	Saccer	S288C	SGD	Goffeau A, et al. Life with 6000 genes. Science 274, 546, 563-567 (1996).
Saccharomycopsis capsularis	Saccap	NRRL Y-17639	Y1000+	This study, Genbank Accession ID: NHAM00000000
Saccharomycopsis fibuligera	Sacfib	KPH12	NCBI	Choo, J. H. et al. Whole-genome de novo sequencing, combined with RNA-Seq analysis, reveals unique genome and physiological features of the amylolytic yeast Saccharomycopsis fibuligera and its interspecies hybrid. Biotechnol. Biofuels 9, 246 (2016).
Saccharomycopsis malanga	Sacmal	JCM 7620	NCBI	Unpublished, Genbank Accession IDs: BCGJ01000001-BCGJ01000044
Saturnispora dispora	Satdis	NRRL Y-1447	Y1000+	This study, Genbank Accession ID: NHAL00000000
Scheffersomyces stipitis	Schsti	CBS 6054	JGI	Jeffries TW, et al. Genome sequence of the lignocellulose-bioconverting and xylose-fermenting yeast Pichia stipitis. Nat. Biotechnol. 25, 319-326 (2007).
Sporopachydermia quercuum	Spoque	JCM 9486	NCBI	Unpublished, Genbank Accession IDs: BCGN01000001-BCGN01000015
Tortispora caseinolytica	Torcas	NRRL Y-17796	JGI	Riley R, et al. Comparative genomics of biotechnologically important yeasts. Proc. Natl. Acad. Sci.

					U.S.A. 113, 9882-9887 (2016).
	Wickerhamomyces anomalus	Wicano	NRRL Y-366-8	JGI	Riley R, et al. Comparative genomics of biotechnologically important yeasts. Proc. Natl. Acad. Sci. U.S.A. 113, 9882-9887 (2016).
	Yarrowia lipolytica	Yarlip	CLIB122	Genolevures	Dujon B, et al. Genome evolution in yeasts. Nature 430, 35-44 (2004).
	Zygosaccharomyces rouxii	Zygrou	CBS 732	Genolevures	Genolevures Consortium, et al. Comparative genomics of protoploid Saccharomycetaceae. Genome Res. 19, 1696-1709 (2009).
Outgroups	*Schizosaccharomyces pombe*	Schpom	972h	JGI	Wood V, et al. The genome sequence of Schizosaccharomyces pombe. Science, (2002)
	Aspergillus nidulans	Aspnid	FGSC A4	JGI	Arnaud MB, et al. The Aspergillus Genome Database (AspGD): recent developments in comprehensive multispecies curation, comparative genomics and community resources. Nucleic Acids Res, (2012); Galagan JE et al. Sequencing of Aspergillus nidulans and comparative analysis with A. fumigatus and A. oryzae. Nature, (2005).

* For *Candida boidinii*, strain GF002 was used for the phylogenomic analysis, and strain NRRL Y-2332 was used for all other analyses including LC-MS/MS cultures. Reference for strain GF002: Borelli G, *et al. De Novo* Assembly of *Candida sojae* and *Candida boidinii* Genomes, Unexplored xylose-consuming yeasts with potential for renewable biochemical production. Genome Announc. 2016;4(1):e01551-15.

**Website**	**URL**
Genolevures	http://www.genolevures.org/download.html# OR http://gryc.inra.fr/
JGI	http://genome.jgi.doe.gov/saccharomycotina/saccharomycotina.info.html
1002YGP	http://1002genomes.u-strasbg.fr/index.html
NCBI	http://www.ncbi.nlm.nih.gov/genome/
SSS	http://www.saccharomycessensustricto.org/cgi-bin/s3.cgi
SGD	http://www.yeastgenome.org/
Y1000+	https://y1000plus.wei.wisc.edu/

Supplementary Note 1

Separate origins of the two tS^{CAG} genes and the tA^{CAG} gene

tRNA^{Ser}(CAG) of the Ser1 clade has been studied extensively in *Candida* species. In this clade it is the product of a single gene or two genes coding for identical tRNAs, and we designate this gene family tS^{CAG}-*A*. (To simplify discussion of tRNA genes, we use a suffix such as -*A* or -*B* to denote each orthogroup of orthologous tRNA genes across different species.) tS^{CAG}-*A* was formed by mutating the anticodon of a gene for a different tRNA^{Ser} isoacceptor, proposed to have been either tS^{AGA} (ref. [11]) or tS^{CGA} (refs. [12,13]). These putative source genes code for tRNAs that translate the 4-codon box of UCN serine codons (Fig. 1). Our phylogenetic analysis (Supplementary Fig. 6) confirms that tS^{CAG}-*A* is monophyletic within the Ser1 clade and that its source was one of the 4-codon box genes, most likely a tS^{AGA} or tS^{UGA} gene, both of which occur in multiple copies in all Ser1 clade species. Conversion of an AGA, UGA or CGA anticodon into CAG by point mutation would require two or three point mutations, and the intermediate steps would cause the tRNA^{Ser} to mistranslate codons for other amino acids which seems maladaptive. However, as previously noted[11,12], the Ser1 clade tS^{CAG}-*A* gene could have been formed from tS^{AGA} or tS^{CGA} in a single step, by inserting a base into the anticodon (AGA → CAGA, or CGA → CAGA). This mutation would change the anticodon sequence to CAG and would not enlarge the anticodon loop if there was an intron in the gene, because the splice donor site would also shift by 1 nucleotide.

In contrast, the tS^{CAG} gene in Ser2 clade species, tS^{CAG}-*B*, is derived from a tS^{GCU} gene coding for the tRNA that translates the 2-codon box of AGY serine codons, as shown by phylogenetic analysis (Supplementary Fig. 7). tS^{CAG}-*B* is more similar to tS^{GCU} than to tS^{AGA}, tS^{UGA} and tS^{CGA}, whereas the opposite is true of tS^{CAG}-*A*. In a phylogenetic tree that includes both of the novel tS^{CAG} genes with some outgroup species, tS^{CAG}-*A* and tS^{CAG}-*B* again cluster with the 4-codon and 2-codon box tRNA genes respectively (Supplementary Fig. 8). Thus, the tS^{CAG} genes of the Ser1 and Ser2 clades have separate evolutionary origins, by mutation of different source genes, which supports the phylogenomic evidence that these clades underwent separate reassignments of CUG from Leu to Ser. tS^{CAG}-*B* is a single-copy gene in all Ser2 clade species except *Ascoidea asiatica* which has two highly similar copies. There are 3-6 tS^{GCU} genes in Ser2 clade species. There is no obvious way to convert a GCU anticodon into a CAG anticodon except by three separate point mutations, which is not possible without intermediate steps in which the mutant tRNA^{Ser} mistranslates codons for at least one other amino acid. The least disruptive route appears to be GCU → GCG → CCG → CAG, by which the tRNA^{Ser} would mistranslate the rare Arg codons CGC and CGG, competing with the normal tRNA^{Arg} molecules.

There is high sequence divergence among the tA^{CAG} genes of the four Ala clade species, but these single-copy genes share a conserved genomic location (Supplementary Fig. 5), therefore they are orthologous. Phylogenetic analysis indicates that tA^{CAG} is derived from a tA^{AGC} gene (not tA^{UGC} as proposed previously[13]), probably by a 1-base insertion into the anticodon loop similar to the mechanism proposed for tS^{CAG}-*A* in *Candida* species[11,12], but a 1-base deletion is also required because the tRNA^{Ala} genes do not contain introns. There are 3-8 tA^{AGC} genes in each Ala clade species, so it is likely that the common ancestor of these species also had a multigene family, of which one member mutated to become tA^{CAG} while the others retained the AGC anticodon.

Supplementary Note 2

<u>Retention of tL^{CAG} as well as tS^{CAG} in the Ser2 clade</u>

All five examined species in the Ser2 clade have the tS^{CAG}-*B* gene that was formed by mutating a tS^{GCU} gene. Four of them also have a second tRNA gene with anticodon CAG, which we infer is a tL^{CAG} gene that has been retained since the common ancestor of the Ser2 and Leu1 clades. It contains several conserved bases characteristic of tRNA^{Leu} including the positions G_{37} and A_{73} that confer Leu rather than Ser identity[1,4] (Supplementary Fig. 9a), and it lacks the multiple G:C basepairs in the extra arm that are characteristic of tRNAs charged with Ser[14]. This gene has a conserved syntenic location beside the protein-coding gene *TRM1* in species of the Ser2 and Leu1 clades (Supplementary Fig. 9b), and the Ser2 and Leu1 tL^{CAG} sequences cluster in a phylogenetic tree (Supplementary Fig. 9c), so we infer that they are orthologs and hence that this gene existed in the common ancestor of the Leu1 and Ser2 clades. We designate this group of orthologous genes tL^{CAG}-*Z*.

Despite the presence of its gene, tRNA^{Leu}(CAG) does not appear to play any significant role in translation in Ser2 clade species. In the peptides sequenced *de novo* from *Saccharomycopsis capsularis* using PEAKS software, 53 genomic CUG (CTG) sites were found to be translated as Ser, and none as Leu (Supplementary Data 3). In peptide mass fingerprinting analysis of the same *S. capsularis* LC-MS/MS data using MaxQuant software, 86 genomic CUG sites were covered by spectra with b- and/or y-ion support. Of these, 78 were translated as Ser, 1 as Leu or Ile, and 7 as other amino acids (Supplementary Table 1). The single detected incorporation of Leu/Ile is similar to the background levels of incorporation of 'incorrect' amino acids seen in other species and at other codons (Supplementary Table 1), occurred in a very short peptide (8 amino acids), and could be explained by many factors including possible error or heterozygosity in the genome sequence. By reverse-transcriptase PCR of RNA samples from *S. capsularis* and *S. malanga* cultures grown in YPD media, we detected transcription of tS^{CAG}-*B* but not tL^{CAG}-*Z* in both species (Supplementary Fig. 10).

However, sequence alignment among the three *Saccharomycopsis* species shows that their tL^{CAG}-*Z* genes are conserved to a greater extent than the surrounding noncoding DNA (Supplementary Fig. 11), which indicates that the gene is being maintained by natural selection and must therefore retain some function. It is possible that *Saccharomycopsis* species require this tRNA in a specific growth condition[15] that we did not examine (for example, meiosis), or even that it is maintained for a function other than translation[16,17]. The fact that tL^{CAG}-*Z* is not present in *Ascoidea rubescens* suggests that its function in Ser2 clade species is not essential.

Supplementary Note 3

<u>Losses of tL^{CAG} and reorganization of CUN-Leu decoding in Leu1 and Leu2 clades</u>
The reassignments of the CUG codon in the Ala, Ser1, and Ser2 clades occurred within a broader context of reorganization of how CUN codons are translated in yeasts. Even among the species that retained the CUG-Leu translation, there have been extensive evolutionary changes in how this translation is achieved, with multiple species losing tL^{CAG} completely and others showing displacement of an ancestral tL^{CAG} by a paralog[13], as summarized in Fig. 4 and described below.

Orthogroups of tL^{CAG} *genes*. By phylogenetic analysis, we identified six orthogroups of tL^{CAG} in Saccharomycotina, which we designated tL^{CAG}-*P*, -*Z*, -*L*, -*T*, -*Q* and -*R*. Each orthogroup consists of a set of tL^{CAG} genes that appear to be orthologs in different species (Supplementary Fig. 9c). Orthogroup tL^{CAG}-*P* is ancestral to the Leu2 clade; orthogroup tL^{CAG}-*Z* is ancestral to the Leu1+Ser2 clades; and orthogroup tL^{CAG}-*L* is ancestral to the Leu0 group of species (Fig. 4). These three orthogroups share a close phylogenetic relationship and are putatively inter-clade orthologs of one another. We refer to them as <u>ancestral</u> tL^{CAG} genes. These ancestral tL^{CAG} genes (*P*, *Z*, and *L*) occur in only one or two copies in the genomes that contain them (Supplementary Fig. 13). The other three orthogroups (*T, Q* and *R*) are not ancestral. Orthogroup tL^{CAG}-*T* is present only in three *Lachancea* (Leu1) species and these genes have a telomeric location. Orthogroups tL^{CAG}-*Q* and -*R* are present only in some Leu2 clade species (Supplementary Figs 9c, 13). The limited phylogenetic distributions of orthogroups *T*, *Q* and *R*, their distant relationship to the ancestral orthogroups *P*, *Z* and *L*, and their presence only in genomes that lack the ancestral genes, suggest that they were probably acquired by horizontal gene transfer.

Three losses of tL^{CAG} *in the Leu1 clade, and one in the Leu2 clade.* Complete loss of all tL^{CAG} genes occurred at least four times in species that retained the standard code (Fig. 4), in addition to the

three losses in clades whose genetic codes changed. In the Leu1 clade, *tL^CAG-Z* was lost in the common ancestor of *Saccharomyces* and *Zygosaccharomyces*, in all *Hanseniaspora* species, and in most species of *Lachancea* (represented by *L. meyersi* in Supplementary Fig. 13). In the Leu2 clade, *tL^CAG-P* was lost in an ancestor of *Candida arabinofermentans*. These species have no $tRNA^{Leu}$(CAG) and instead probably read CUG by wobble using $tRNA^{Leu}$(UAG) with an unmodified U_{34} base as occurs in *S. cerevisiae*[18]. Other species in the Leu1 and Leu2 clades lost their ancestral *tL^CAG-Z* or *tL^CAG-P* gene but replaced it by acquiring a paralogous *tL^CAG* from a different orthogroup: *tL^CAG-T* in *L. thermotolerans* and two closely related *Lachancea* species, and *tL^CAG-Q* and *tL^CAG-R* in the common ancestor of many Leu2 clade species including *Pichia kudriavzevii* (Supplementary Figs 9c, 13).

Intron expansion in Leu1 clade tL^CAG. The losses of *tL^CAG-Z* in the Leu1 clade appear to have been preceded by an extraordinary expansion of the intron in this gene. tRNA introns are typically short; the interquartile range among 3,723 tRNA introns in the yeast species we studied is 15–31 nt. However, the introns of *tL^CAG-Z* in the genera *Kluyveromyces* and *Eremothecium,* and in *Lachancea kluyveri* (the only *Lachancea* species that retains the ancestral gene), range from 134–318 nt, making them the longest canonical tRNA introns known in yeasts (Supplementary Fig. 12) or any eukaryote[19]. The long introns contain extensive predicted secondary structure that may slow the rate of formation of the mature spliced and base-modified tRNA. Expansion of the intron may have been a response to a killer toxin, because the intron is located in the anticodon loop. Anticodon nucleases recognize the anticodon loop and are unlikely to cleave pre-tRNAs until after splicing occurs, and in some cases also require base modification of the tRNA[20-23]. In the standard numbering system for base positions in tRNAs, the anticodon is at positions 34-36, introns are located between positions 37 and 38, and killer toxin nucleases cleave between positions 34 and 35 within the anticodon.

Reorganization of CUN codon:anticodon wobble in the Leu1 clade. Across the whole genetic code table, most yeast species use similar repertoires of tRNA anticodons for translation[24,25], even though they can vary widely in the number of genes for different isoacceptors. The last common ancestor of Saccharomycotina translated CUN as Leu using three $tRNA^{Leu}$ isoacceptors with anticodons AAG (modified to IAG, decoding CUU and CUC codons), UAG (decoding CUA), and CAG (decoding CUG). This configuration is present in the paraphyletic Leu0 group of species which includes the root of the tree (Fig. 2; Supplementary Fig. 13). However, the Leu1 clade (excluding *Cyberlindnera* and *Wickerhamomyces*) underwent three evolutionary changes to the wobble arrangements for CUN-Leu decoding:

- The 'tRNA sparing' rule[24] was broken. Eukaryotes almost invariably use anticodons with A_{34} rather than G_{34} to read NYY codons[24]. In other words they use anticodon AAG (modified to IAG) rather than GAG to read the Leu codons CUU and CUC, whereas bacteria

do the opposite. This is true of most Saccharomycotina, but a switch to the bacterial pattern occurred in the main part of the Leu1 clade after it separated from *Cyberlindnera* and *Wickerhamomyces*.

- In the genera *Naumovozyma* and *Kazachstania* the normal eukaryotic 'tRNA sparing' pattern was subsequently reinstated, by losing tL^{GAG} and making new tL^{AAG} genes.
- In *Hanseniaspora vineae* and *H. osmophila*, tL^{GAG} was lost and $tRNA^{Leu}$(UAG) now reads all four CUN codons.

Supplementary References

1 Giegé, R., Sissler, M. & Florentz, C. Universal rules and idiosyncratic features in tRNA identity. *Nucleic Acids Res.* **26**, 5017-5035 (1998).

2 Gordon, J. L., Byrne, K. P. & Wolfe, K. H. Additions, losses and rearrangements on the evolutionary route from a reconstructed ancestor to the modern *Saccharomyces cerevisiae* genome. *PLoS Genet.* **5**, e1000485 (2009).

3 Suzuki, T., Ueda, T. & Watanabe, K. The 'polysemous' codon--a codon with multiple amino acid assignment caused by dual specificity of tRNA identity. *EMBO J.* **16**, 1122-1134 (1997).

4 Huang, Q., Yao, P., Eriani, G. & Wang, E. D. In vivo identification of essential nucleotides in tRNALeu to its functions by using a constructed yeast tRNALeu knockout strain. *Nucleic Acids Res.* **40**, 10463-10477 (2012).

5 Johansson, M. J. & Bystrom, A. S. Dual function of the tRNA (m(5)U54) methyltransferase in tRNA maturation. *RNA* **8**, 324-335 (2002).

6 Arimbasseri, A. G., Blewett, N. H., Iben, J. R., Lamichhane, T. N., Cherkasova, V., Hafner, M. & Maraia, R. J. RNA polymerase III output is functionally linked to tRNA dimethyl-G26 modification. *PLoS Genet.* **11**, e1005671 (2015).

7 Simao, F. A., Waterhouse, R. M., Ioannidis, P., Kriventseva, E. V. & Zdobnov, E. M. BUSCO: assessing genome assembly and annotation completeness with single-copy orthologs. *Bioinformatics* **31**, 3210-3212 (2015).

8 Shimodaira, H. An approximately unbiased test of phylogenetic tree selection. *Syst. Biol.* **51**, 492-508 (2002).

9 Shimodaira, H. & Hasegawa, M. Multiple comparisons of log-likelihoods with applications to phylogenetic Inference. *Mol. Biol. Evol.* **16**, 1114-1116 (1999).

10 Fukuhara, H. Linear DNA plasmids of yeasts. *FEMS Microbiol. Lett.* **131**, 1-9 (1995).

11 Yokogawa, T., Suzuki, T., Ueda, T., Mori, M., Ohama, T., Kuchino, Y., Yoshinari, S., Motoki, I., Nishikawa, K., Osawa, S. & et al. Serine tRNA complementary to the nonuniversal serine codon CUG in *Candida cylindracea*: evolutionary implications. *Proc. Natl. Acad. Sci. USA* **89**, 7408-7411 (1992).

12 Massey, S. E., Moura, G., Beltrao, P., Almeida, R., Garey, J. R., Tuite, M. F. & Santos, M. A. Comparative evolutionary genomics unveils the molecular mechanism of reassignment of the CTG codon in *Candida* spp. *Genome Res.* **13**, 544-557 (2003).

13 Mühlhausen, S., Findeisen, P., Plessmann, U., Urlaub, H. & Kollmar, M. A novel nuclear genetic code alteration in yeasts and the evolution of codon reassignment in eukaryotes. *Genome Res.* **26**, 945-955 (2016).

14 Moura, G. R., Paredes, J. A. & Santos, M. A. Development of the genetic code: insights from a fungal codon reassignment. *FEBS Lett.* **584**, 334-341 (2010).

15 Prat, L., Heinemann, I. U., Aerni, H. R., Rinehart, J., O'Donoghue, P. & Soll, D. Carbon source-dependent expansion of the genetic code in bacteria. *Proc. Natl. Acad. Sci. USA* **109**, 21070-21075 (2012).

16 Murray, L. E., Rowley, N., Dawes, I. W., Johnston, G. C. & Singer, R. A. A yeast glutamine tRNA signals nitrogen status for regulation of dimorphic growth and sporulation. *Proc. Natl. Acad. Sci. USA* **95**, 8619-8624 (1998).

17 Katz, A., Elgamal, S., Rajkovic, A. & Ibba, M. Non-canonical roles of tRNAs and tRNA mimics in bacterial cell biology. *Mol. Microbiol.* **101**, 545-558 (2016).

18 Johansson, M. J., Esberg, A., Huang, B., Bjork, G. R. & Bystrom, A. S. Eukaryotic wobble uridine modifications promote a functionally redundant decoding system. *Mol. Cell. Biol.* **28**, 3301-3312 (2008).

19 Yoshihisa, T. Handling tRNA introns, archaeal way and eukaryotic way. *Front. Genet.* **5**, 213 (2014).

20 Lu, J., Huang, B., Esberg, A., Johansson, M. J. & Bystrom, A. S. The *Kluyveromyces lactis* gamma-toxin targets tRNA anticodons. *RNA* **11**, 1648-1654 (2005).

21 Satwika, D., Klassen, R. & Meinhardt, F. Anticodon nuclease encoding virus-like elements in yeast. *Appl. Microbiol. Biotechnol.* **96**, 345-356 (2012).

22 Chakravarty, A. K., Smith, P., Jalan, R. & Shuman, S. Structure, mechanism, and specificity of a eukaryal tRNA restriction enzyme involved in self-nonself discrimination. *Cell Reports* **7**, 339-347 (2014).

23 Hopper, A. K. Transfer RNA post-transcriptional processing, turnover, and subcellular dynamics in the yeast *Saccharomyces cerevisiae*. *Genetics* **194**, 43-67 (2013).

24 Marck, C., Kachouri-Lafond, R., Lafontaine, I., Westhof, E., Dujon, B. & Grosjean, H. The RNA polymerase III-dependent family of genes in hemiascomycetes: comparative RNomics, decoding strategies, transcription and evolutionary implications. *Nucleic Acids Res.* **34**, 1816-1835 (2006).

25 Kollmar, M. & Mühlhausen, S. How tRNAs dictate nuclear codon reassignments: Only a few can capture non-cognate codons. *RNA Biol.* **14**, 293-299 (2017).
