# Supplementary material for: Evolutionary instability of CUG-Leu in the genetic code of budding yeasts
Source: Nat Commun. 2018 May 14;9:1887. doi: 10.1038/s41467-018-04374-7 (PMC5951914; doi:10.1038/s41467-018-04374-7)
Supplement: Supplementary file 2 — Description of Additional Supplementary Files [file 41467_2018_4374_MOESM2_ESM.pdf]

Description of Additional Supplementary Files

File Name: Supplementary Data 1
Description: BLAST-based predictions and mass spectrometry validations of genetic codes. Histograms show the frequencies of amino acid matches to CUG codons in BLAST analyses (left columns), and LC/MS-MS peptide mass fingerprinting where conducted (right columns), for species in each clade: **(a)** Ala clade; **(b,c)** Ser1 clade; **(d)** Ser2 clade; **(e,f)** Leu0 species; **(g,h)** Leu1 clade; **(i,j)** Leu2 clade. For BLAST-based predictions, the scores on the Y-axis represent the number of CUG codon sites in the genome that align to amino acid residues in the YGOB protein database. For LC-MS/MS experiments, the scores on the Y-axis represent the number of CUG codon sites in the genome that align to residues with b- or y-ion support in peptides identified by mass fingerprinting using MaxQuant. **(PDF file, 10 pages).**

File Name: Supplementary Data 2
Description: BLAST-based predictions of genetic codes in 54 species. Each file contains a matrix of 64 codons x 20 amino acids for one species. Each cell in the matrix shows the count of the number of times the codon was aligned opposite the amino acid in BLASTP searches of genes from the query species versus a database of proteins from the YGOB database (all of which use the standard genetic code), scaled so that the total score for any codon site with hits is 1. Orange cells indicate the standard genetic code, and (where applicable) other colors indicate non-standard translations with high scores. Only codons that were aligned opposite the same amino acid in more than 80% of the BLASTP HSPs were scored. Columns on the right for each codon are: Sum, total number of instances of this codon (in the ORFs in the genome) that lie within alignments (BLAST HSPs) to proteins in the database; Universal %, number of instances of this codon that were aligned to its standard translation, as a percentage of the Sum; Largest %, number of instances of this codon that were aligned to the most common translation, as a percentage of the Sum; All codons, number of instances of this codon in the ORFs of this genome. **(Zip archive of 54 Excel files).**

File Name: Supplementary Data 3
Description: Genetic codes determined in 18 species by *de novo* peptide sequencing. Each file contains a matrix of 64 codons x 19 amino acids (Leu and Ile are not distinguishable) for one species. Each cell in the matrix shows the count of the number of times the codon was aligned opposite the amino acid, in alignment of a *de novo* sequenced peptide from the LC/MS-MS experiment versus genes from the query species, scaled so that the total score for any codon site (in the genome) with hits is 1. Orange cells indicate the standard genetic code, and (where applicable) other colors indicate non-standard translations with high scores. Only codons that were aligned opposite the same amino acid in all matching *de novo* peptides were scored. Columns on the right for each codon are: Sum, total number of instances of this codon (in the ORFs in the genome) that lie within alignments to *de novo* peptides; Universal %, number of instances of this codon that were aligned to its standard translation, as a percentage of the Sum; Largest %, number of instances of this codon that were aligned to the most common translation, as a percentage of the Sum; All codons, number of instances of this codon in the ORFs of this genome. **(Zip archive of 18 Excel files).**

File Name: Supplementary Data 4
Description: Parameter settings for MaxQuant analyses. **(text file).**
