# Supplementary material for: Evolutionary instability of CUG-Leu in the genetic code of budding yeasts
Source: Nat Commun. 2018 May 14;9:1887. doi: 10.1038/s41467-018-04374-7 (PMC5951914; doi:10.1038/s41467-018-04374-7)
Supplement: Supplementary file 3 — Supplementary Data 1 [file 41467_2018_4374_MOESM3_ESM.pdf]

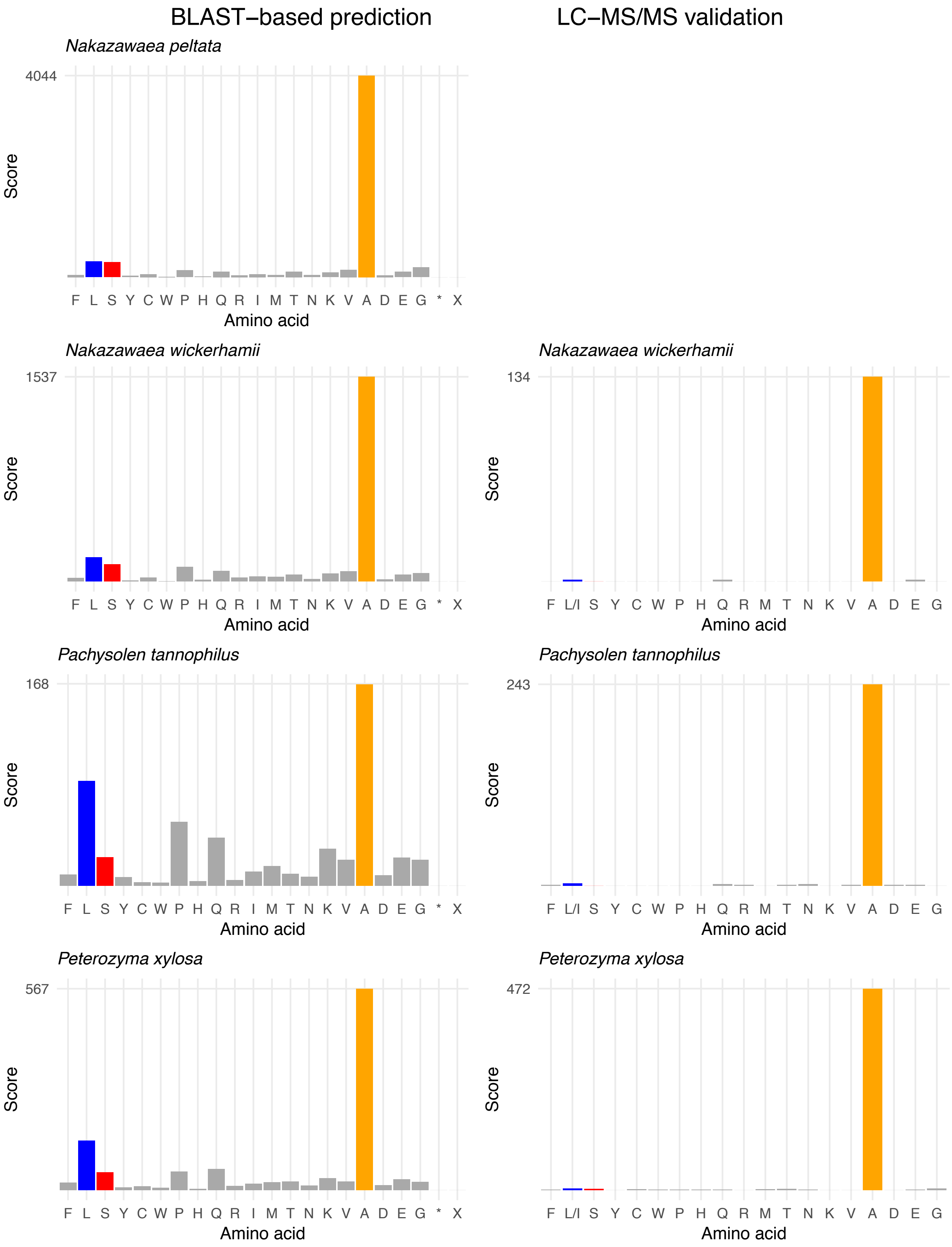
BLAST−based prediction
LC−MS/MS validation
Nakazawaea peltata
4044
Score
F L S Y C W P H Q R I M T N K V A D E G * X
Amino acid
Nakazawaea wickerhamii
1537
Score
F L S Y C W P H Q R I M T N K V A D E G * X
Amino acid
Nakazawaea wickerhamii
134
Score
F L/I S Y C W P H Q R M T N K V A D E G
Amino acid
Pachysolen tannophilus
168
Score
F L S Y C W P H Q R I M T N K V A D E G * X
Amino acid
Pachysolen tannophilus
243
Score
F L/I S Y C W P H Q R M T N K V A D E G
Amino acid
Peterozyma xylosa
567
Score
F L S Y C W P H Q R I M T N K V A D E G * X
Amino acid
Peterozyma xylosa
472
Score
F L/I S Y C W P H Q R M T N K V A D E G
Amino acid

(a) Ala clade

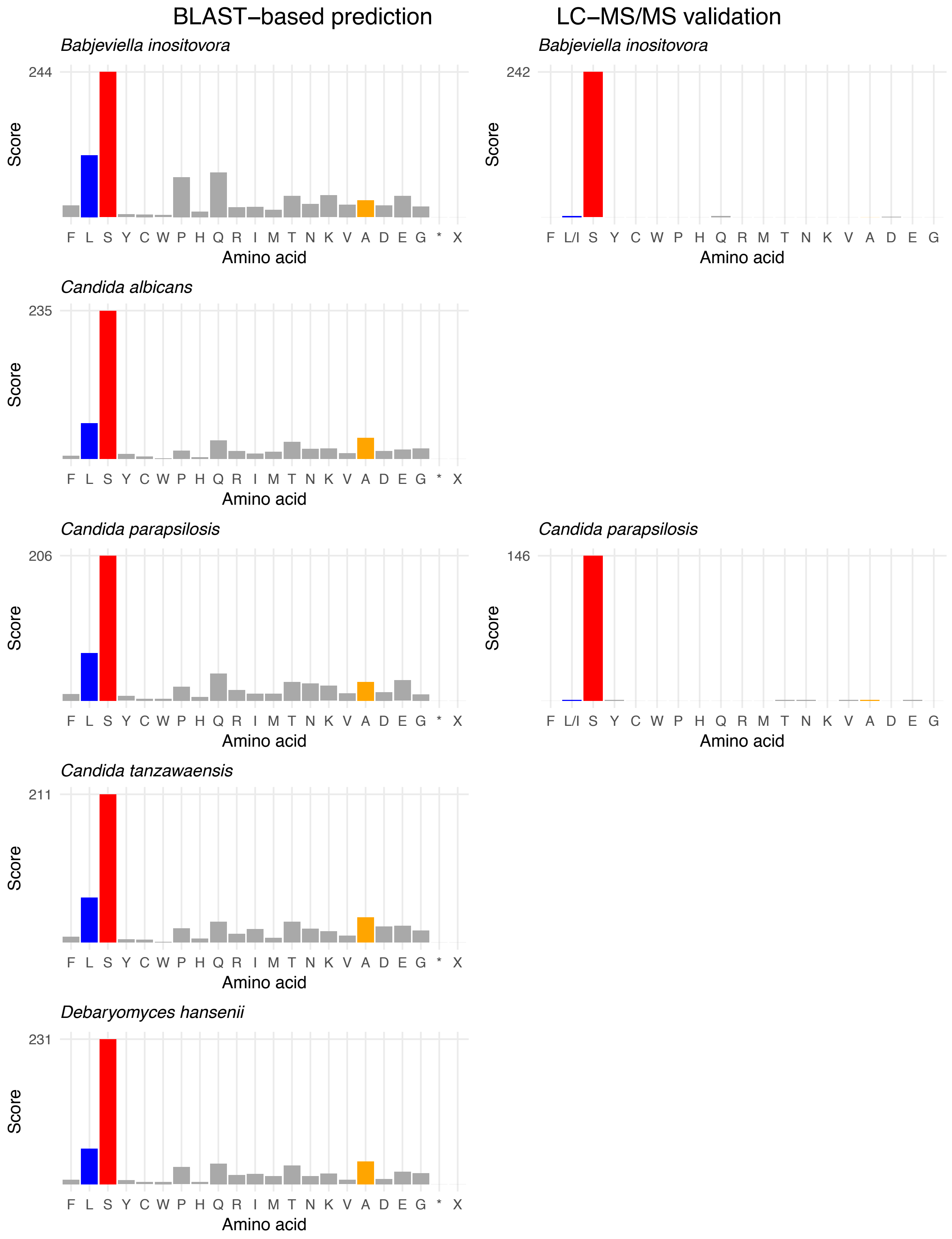

(b) Ser1 clade (page 1)

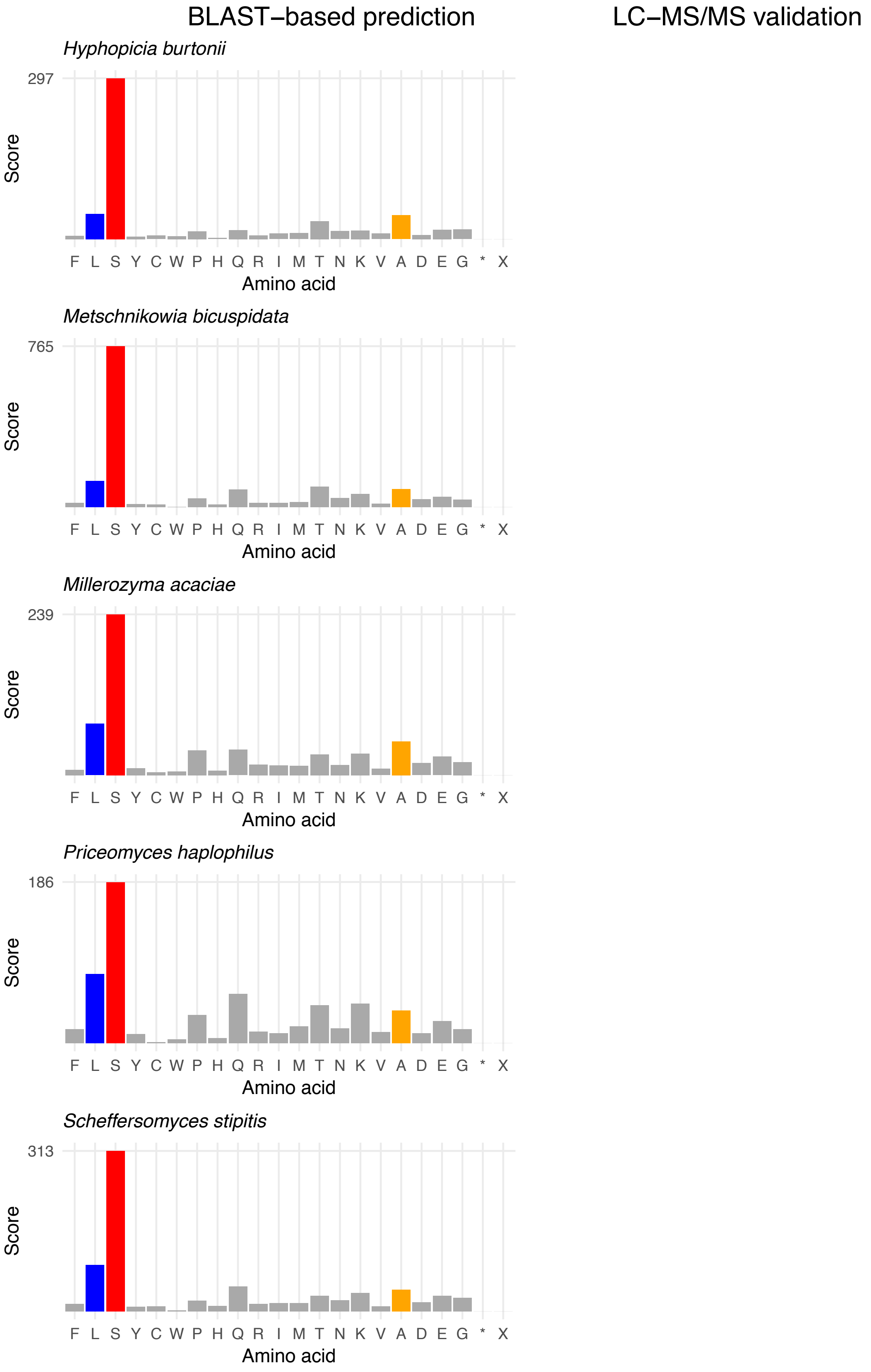

(c) Ser1 clade (page 2)

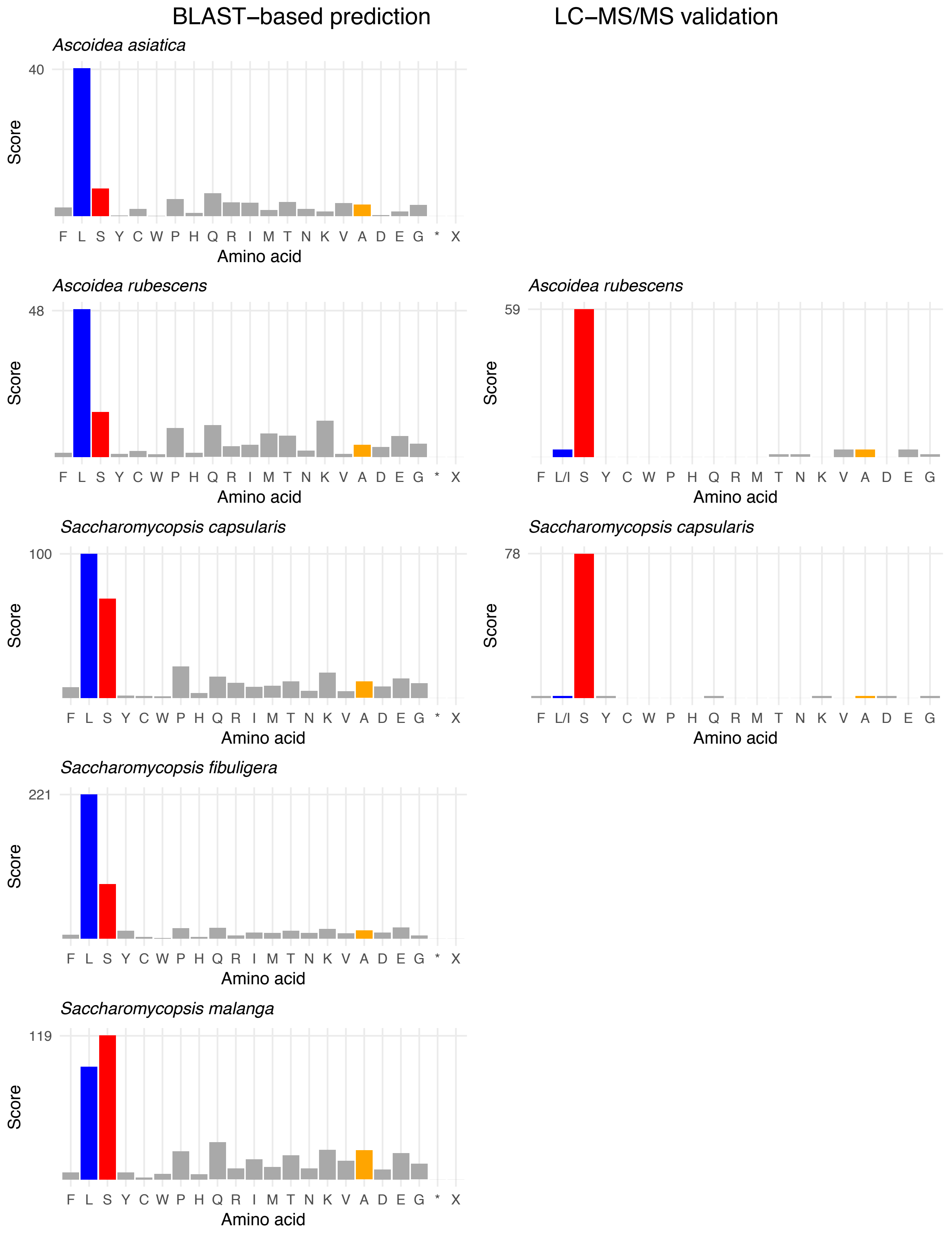
BLAST−based prediction
LC−MS/MS validation
Ascoidea asiatica
Ascoidea rubescens
Saccharomycopsis capsularis
Saccharomycopsis fibuligera
Saccharomycopsis malanga
Score
Amino acid
40
48
59
100
78
221
119
F L S Y C W P H Q R I M T N K V A D E G * X
F L/I S Y C W P H Q R M T N K V A D E G

(d) Ser2 clade

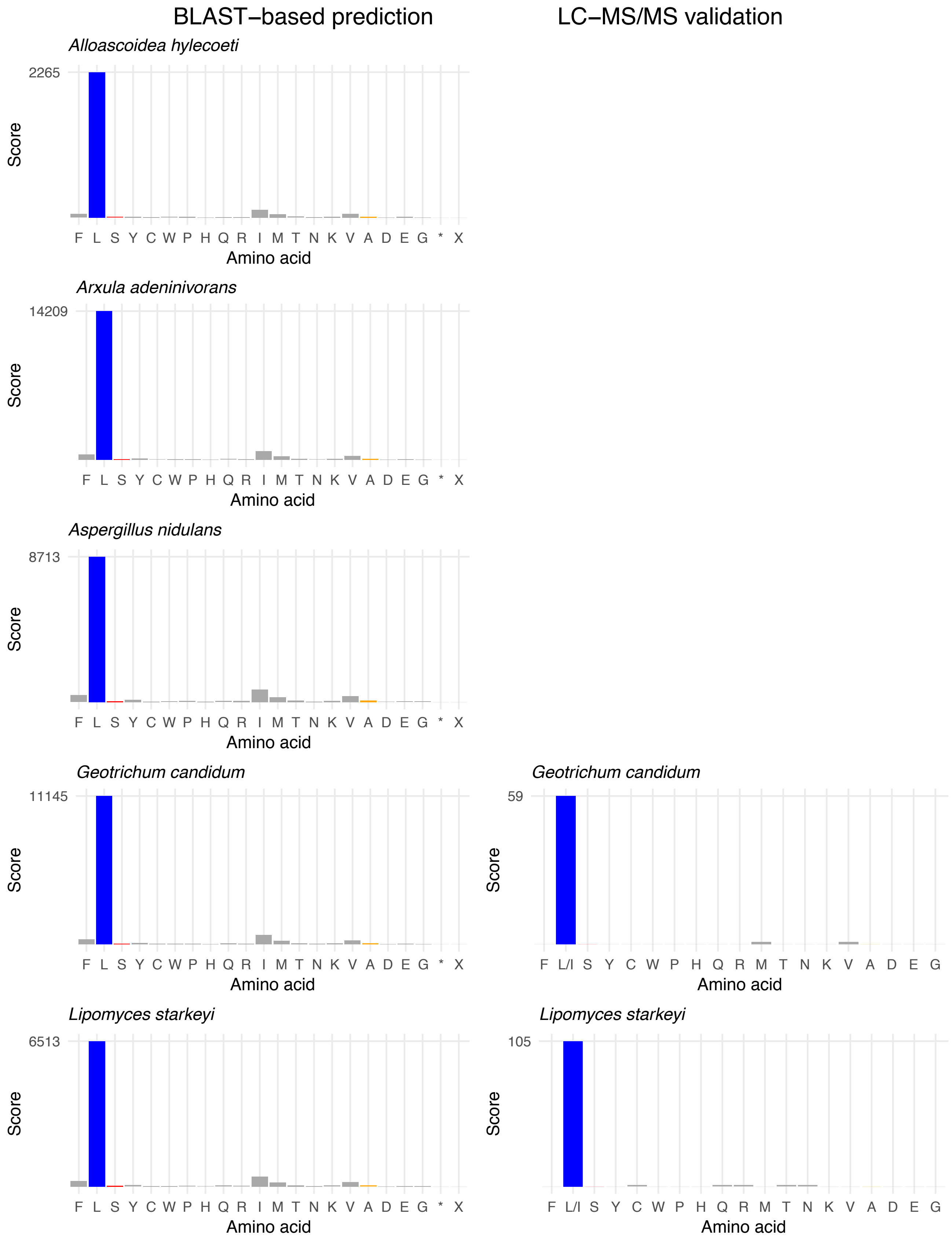

(e) Leu0 species (page 1)

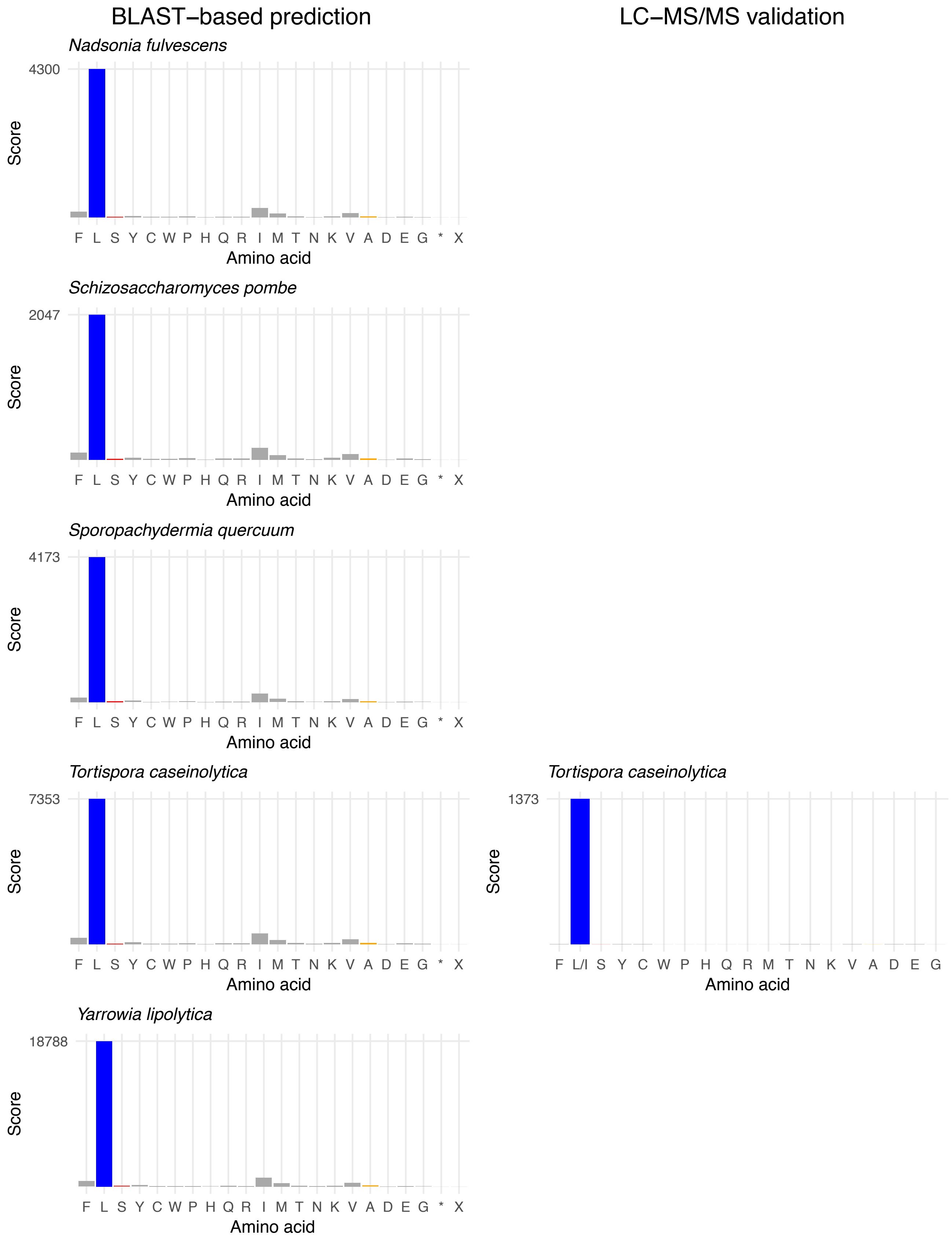

(f) Leu0 species (page 2)

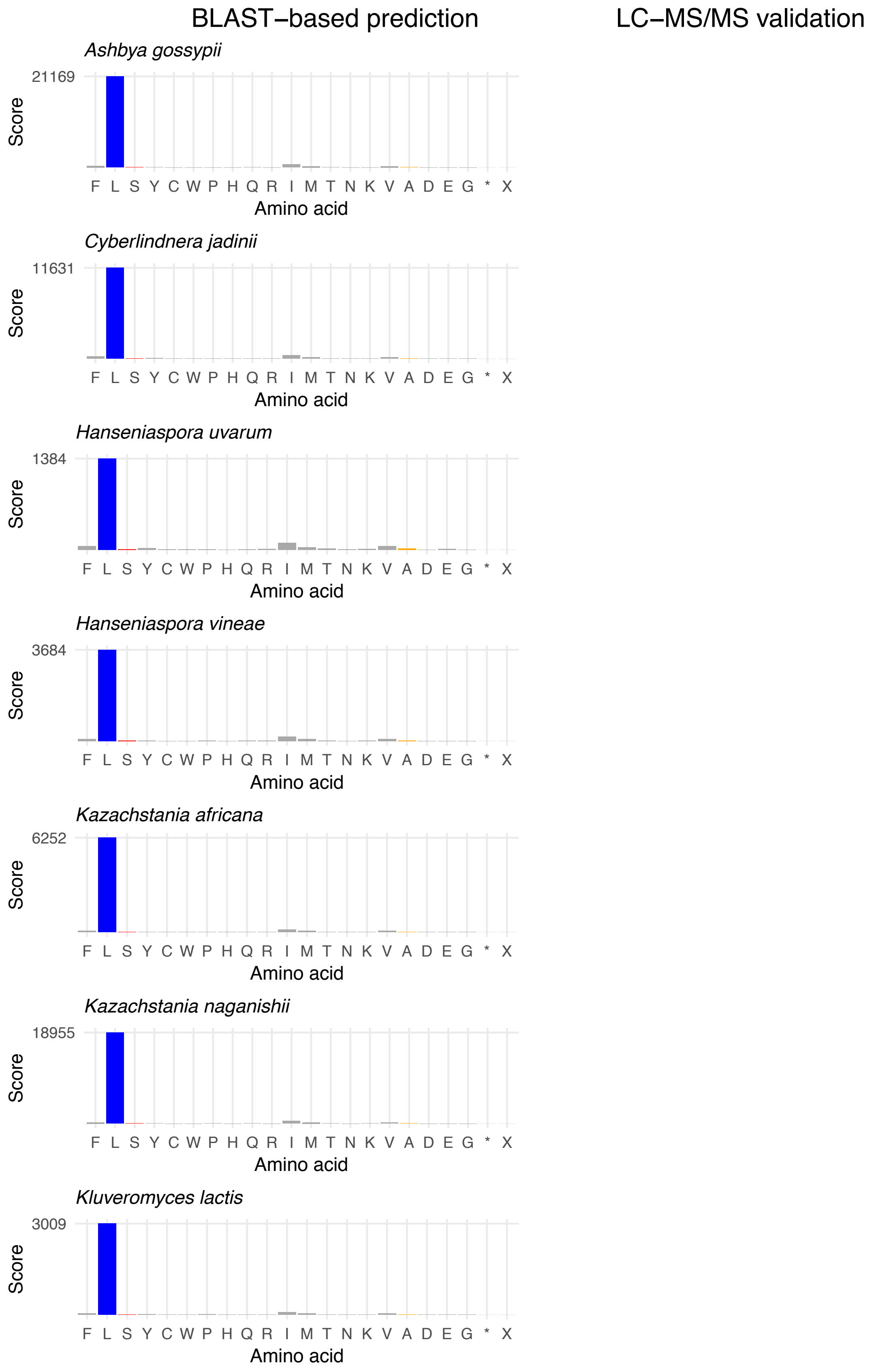

(g) Leu1 clade (page 1)

BLAST−based prediction

LC−MS/MS validation

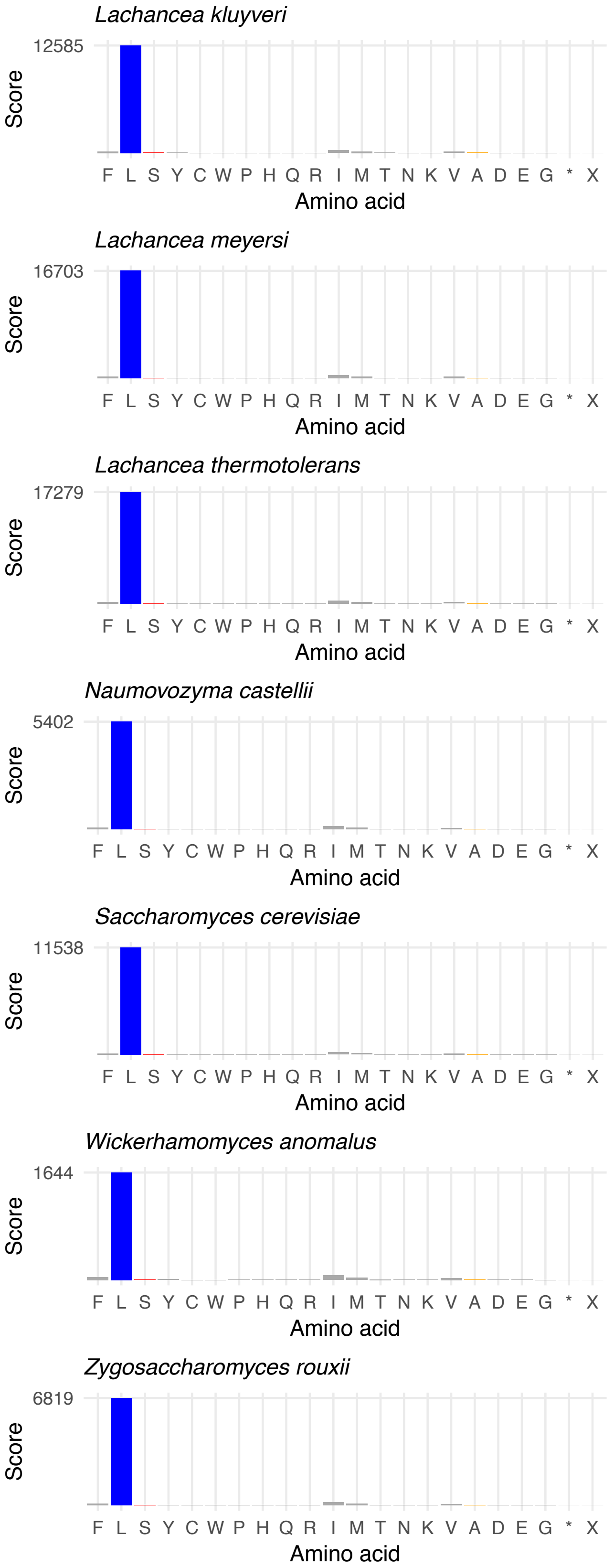

(h) Leu1 clade (page 2)

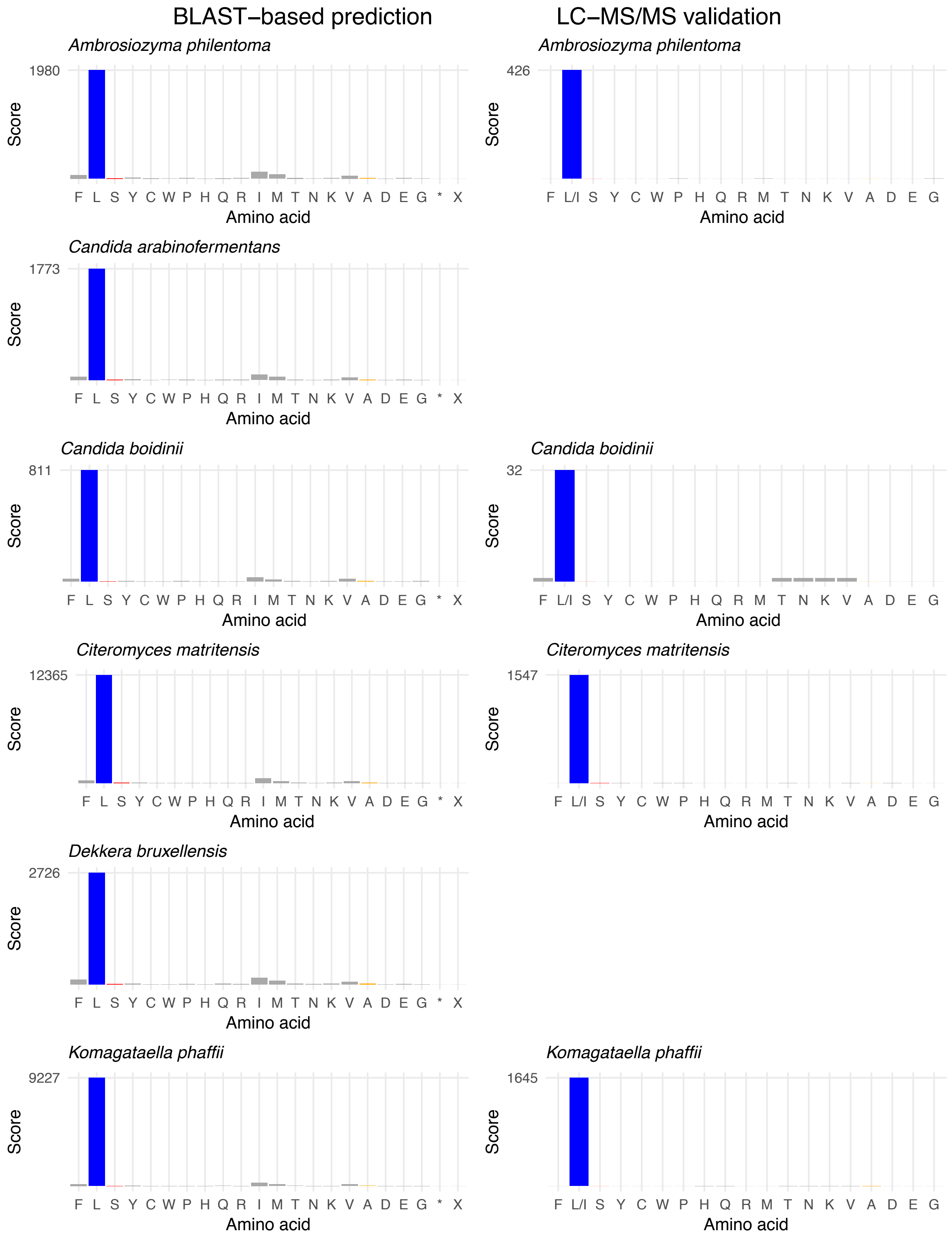

(i) Leu2 clade (page 1)

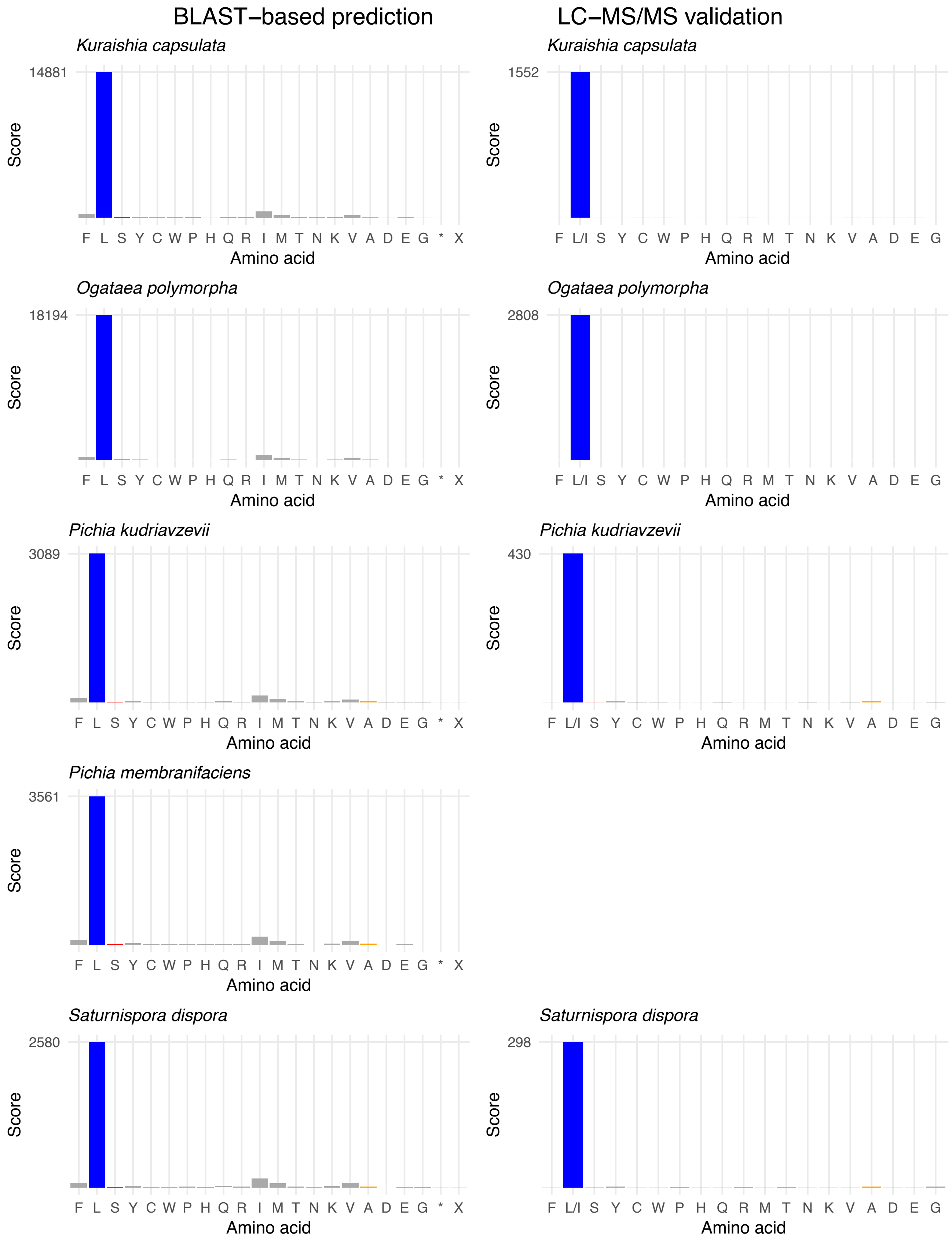

(j) Leu2 clade (page 2)
